# *MicroRNA-3906* Regulates Fast Muscle Differentiation through Modulating the Target Gene *homer-1b* in Zebrafish Embryos

**DOI:** 10.1371/journal.pone.0070187

**Published:** 2013-07-31

**Authors:** Cheng-Yung Lin, Jie-Shin Chen, Moo-Rung Loo, Chung-Ching Hsiao, Wen-Yen Chang, Huai-Jen Tsai

**Affiliations:** Institute of Molecular and Cellular Biology, National Taiwan University, Taipei, Taiwan; UMR CNRS 5242 - ENS de Lyon- Université Lyon 1, France

## Abstract

A microRNA, termed *miR-In300* or *miR-3906*, suppresses the transcription of *myf5* through silencing *dickkopf-related protein 3* (*dkk3r/dkk3a*) during early development when *myf5* is highly transcribed, but not at late stages when *myf5* transcription is reduced. Moreover, after 24 hpf, when muscle cells are starting to differentiate, Dkk3a could not be detected in muscle tissue at 20 hpf. To explain these reversals, we collected embryos at 32 hpf, performed assays, and identified *homer-1b*, which regulates calcium release from sarcoplasmic reticulum, as the target gene of *miR-3906*. We further found that either *miR-3906* knockdown or *homer-1b* overexpression increased expressions of *fmhc4* and *atp2a1* of calcium-dependent fast muscle fibrils, but not slow muscle fibrils, and caused a severe disruption of sarcomeric actin and Z-disc structure. Additionally, compared to control embryos, the intracellular calcium concentration ([Ca^2+^]_i_) of these treated embryos was increased as high as 83.9–97.3% in fast muscle. In contrast, either *miR-3906* overexpression or *homer-1b* knockdown caused decreases of [Ca^2+^]_i_ and, correspondingly, defective phenotypes in fast muscle. These defects could be rescued by inducing *homer-1b* expression at later stage. These results indicate that *miR-3906* controls [Ca^2+^]_i_ homeostasis in fast muscle through fine tuning *homer-1b* expression during differentiation to maintain normal muscle development.

## Introduction

MicroRNAs (miRNAs) are short (19–22 nt) endogenous non-coding RNAs that regulate gene expression at the post-transcriptional level by binding the seed sequence(s) located at the 3′-untranslated region (3′UTR) of target mRNA(s), resulting in degradation, deadenylation or activation of target mRNA(s) [1]–[3]. Therefore, miRNAs play important roles in translational control by fine tuning the protein level to reach a dynamic equilibrium in cells.

In vertebrates, the regulatory mechanisms of many myogenic genes involved in muscle development at the transcriptional level have been studied [4]. Skeletal muscle development is mainly controlled by myogenic regulatory factors (MRFs), including *myogenic factor 5* (*myf5*), *myoblast determination protein* (*myod*), *muscle-specific regulatory factor 4* (*mrf4*; also known as *myf6*) and *myogenin* (*myog*), which enable activation of muscle-specific gene expression [5]. Several muscle-specific miRNAs were found in higher vertebrates, and it is known that these miRNAs are involved in muscle development at the translational level [6]. For example, the main function of *miR-1*, *miR-133* and *miR-206* is inhibition of their target genes in order to promote differentiation of muscle cells [7]–[9]. Both *miR-208b* and *miR-499*, which are expressed in skeletal muscles, regulate the specification of muscle fiber identity by activating slow myofiber genes, while repressing fast myofiber genes [10]. The target genes for zebrafish *miR-1* and *miR-133* are involved in actin-binding, as well as actin-related and vesicular transport [11]. Absence of *miR-1* and *miR-133* by co-injection of mopholinos (MOs) into embryos disturbs the organization of actin in muscle fibers. The expressions of *miR-1*, *miR-133*, *miR-206* and *miR-208* in muscle cells are mainly controlled by MRFs, serum response factor (SRF) and MEF2 [12]–[15].

In a previous study, we identified a 300-nucleotide repressive *cis*-element located at the first intron of zebrafish *myf5,* which is able to inhibit the promoter activity of *myf5* [16]. Upon further study, we uncovered a novel miRNA, termed *miR-3906,* or *miR-In300*, which exists in this intron [17]. During early embryonic muscle development, we used our Labeled miRNA pull-down (LAMP) assay [18] to search for the putative target genes of *miR-3906* from extracts of embryos at 16 hpf when *myf5* expression reaches its highest expression during embryogenesis [19]. We found that *dickkopf-related protein 3* (*dkk3r/dkk3a*) is the target gene of *miR-3906* [17]. Dkk3a is an activator which controls the ability of zebrafish *myf5* promoter to affect trunk muscle development through phosphorylated p38a-dependent Smad4 activity [20]. However, in mature trunk somites, *miR-3906* silences Dkk3a production through binding the 3′UTR of *dkk3a* mRNA, resulting in the decrease of *myf5* promoter activity and, hence, the reduction of Myf5 in mature somites [17]. Therefore, the intronic element of host gene *myf5* produces *miR-3906* to silence the upstream regulator Dkk3a, which, in turn, serves as a negative modulator of control.

Based on whole-mount *in situ* hybridization (WISH) analysis of embryos at 20 hpf, *myf5* is expressed in the newly forming somites, but absent in mature somites. On the other hand, *miR-3906* is absent in the newly forming somites, but present in mature somites, gradually enhancing its expression as somites mature [17]. This finding indicates that *miR-3906* is derived from the *myf5* transcripts in somites. In a previous study, we used LAMP to search for the target gene of *miR-3906* in 16-hpf embryos when the muscle cells are at the stages of specification and proliferation. However, at the later developmental stages, e.g., after 24 hpf, when muscle cells are starting to differentiate, we noticed that Dkk3a could not be detected in muscle tissue at 20 hpf [17]. Since the promoter activity of *myf5* is no longer activated by Dkk3a, *myf5* is greatly reduced in the trunk, resulting in the differentiation of muscle cells. Thus, when muscle cells are at the differentiation stage, we do not know (a) the status of *miR-3906* expression in somites, (b) the target gene of *miR-3906*, or (c) the biological function of *miR-3906* in the context of its target gene during late developmental stage.

In general, target genes that are predicted, either from bioinformatics methods or from cell lines, cannot be easily attached to particular developmental stages. Therefore, to learn how *miR-3906* functions in muscle development at late stage through controlling the target gene, we collected embryos at late stage, such as 32 hpf, when muscle development is at the differentiation stage, and performed a LAMP assay combined with Zebrafish Whole Genome Microarray to obtain putative target genes of *miR-3906.* We identified *homer-1b*, which is a scaffolding protein that binds ryanodine receptor to regulate calcium release from sarcoplasmic reticulum [21], as a target of *miR-3906* at late stage in embryos. While *dkk3a* had previously been identified as the target gene of *miR-3906* at early developmental stage [17], assays performed on embryos collected at 32 hpf indicated that both *miR-3906* and *homer-1b* are expressed in mature somites and that their presence affects the expressions of *fmhc4* and *atp2a1* of calcium-dependent fast muscle fibrils, but not slow muscle fibrils. Finally, *miR-3906* was found to control homeostasis of intracellular calcium concentration ([Ca^2+^]_i_) in fast muscle fibrils during differentiation through fine tuning *homer-1b* expression.

## Materials and Methods

### Fish Embryos

The wild-type zebrafish (*Danio rerio*) AB strain (University of Oregon, Eugene, OR) and the transgenic line Tg(α-actin:RFP) [22] were used. They were cultured at 28.5°C under a light and dark cycle of 14 and 10 hours, respectively. Production and stage identification of embryos followed the description by Westerfield [23] and Kimmel *et al*. [24]. For drug treatment, WT embryos at 20 hpf were moved from clean water and soaked in water containing 150 ppm caffeine or 10 µM 2-APB for four hr. The fluorescence signal was observed under a fluorescent stereomicroscope MZ FLIII (Leica).

### Ethics Statement

The National Taiwan University Institutional Animal Care and Use Committee (IACUC) reviewed and approved the protocol described below (NTU-99-72). No specific ethics approval was required for this project, as all zebrafish (*Danio rerio*) used in this study were between 0 to 3 days old. This procedure is not considered painful since embryos at this early stage have no pain perception.

### Searching for the Putative Target Genes of *miR-3906*

To search for the putative target genes of *miR-3906*, we performed a LAMP assay [18] with some modifications. The pre-*miR-3906* was labeled with Biotin and then mixed with cell extracts. The putative target genes were precipitated by anti-Biotin agarose beads (Sigma) and transformed into cDNA by reverse transcriptase-polymerase chain reaction. Finally, these putative cDNAs for *miR-3906-*targeting were further combined with Zebrafish Whole Genome Microarray (Aligent).

### Plasmid Constructs

Based on the NCBI database, we designed primers to perform PCR from the 32 hpf cDNA library to obtain the complete 3′UTR segments of each cDNA of *colla2* (NM182968, +3963 to +4965), *dnajc10* (NM001083547, +2377 to +2733), *homer-1b* (NM001002496, +1198 to +2222), *six1a* (NM207095, +856 to +1117), and *trmt2a* (NM199929, +1861 to +2011). Each PCR product was ligated into the downstream of luciferase (*luc*) gene in plasmid phRG-TK and designated as plasmid phRG-TK-*col1a2*-3′UTR, -*dnajc10*-3′UTR, -*homer-1b*-3′UTR, -*six1a*-3′UTR and -*trmt2a*-3′UTR, respectively. The 3′UTR sequence of each of the five genes was driven by thymidine kinase (TK) promoter.

Plasmids pHsp-*miR-3906* and pHsp-*wob-homer-1b* contain pre-*miR-3906* RNA and wobble *homer-1b* mRNA, respectively, driven by heat-shock treatment. Wobble *homer-1b* mRNA is a mutated form derived from *homer-1b* mRNA, in which the target sequences of *homer-1b*-MO are mutated, and the 3′UTR of *homer-1b* mRNA is not included, effectively eliminating targets for both *miR-3906* and *homer-1b*-MO. Using microinjection of plasmids pHsp-*miR-3906* and pHsp-*wob-homer-1b* in embryos, overexpression of *miR-3906* RNA and wobble *homer-1b* mRNA, respectively, was generated at 20 hpf by heat-shock induction.

### Validation of *miR-3906*-targeting Genes by *luc* Activity Assay

Dual *luc* reporter assay (Promega) was carried out in cell line HEK-293T and zebrafish embryos by following the method described previously [25] with some modifications. In control group, we co-transfected 40 ng of plasmid pGL3-TK, which served as an internal control, and 200 ng of each examined plasmid, including phRG-TK-*col1a2*-3′UTR, phRG-TK-*dnajc10*-3′UTR, phRG-TK-*homer-1b*-3′UTR, phRG-TK-*six1a*-3′UTR, and phRG-TK-*trmt2*-3′UTR. The *luc* activity of the control group was set 100%. In the experimental group, we co-transfected 40 ng of pGL3-TK, 200 ng of each examined plasmid and 2 µg of plasmid pCMV-RFP-*miR-3906.* In zebrafish embryos, we co-injected 5 ng/µl of pGL3-TK, which also served as an internal control, and 5 ng/µl of each examined plasmid as described above to serve as a control group; whereas we co-injected 5 ng/µl of pGL3-TK, 5 ng/µl of each examined plasmid and 200 pg of synthesized pre-*miR-3906* RNA to serve as an experimental group. All plasmids were microinjected into one-cell embryos in a volume of 2.3 nl. Twenty embryos were harvested for *luc* assay after injection for 24 hr. The change of *luc* activity was calculated as follows: Fold change = [(Renilla *luc*+*miR-3906*)/(Firefly *luc*+*miR-3906*)] ÷ [(Renilla *luc*)/(Firefly *luc*)].

### Whole-mount in situ Hybridization (WISH)

WISH followed the method described previously by Lee *et al*. [26], except that anti-sense sequences of *miR-3906* (AAAATCTGCATTCAAAATGCTTT), *miR-206* (CCACACACTTCCTTACATTCCT) and control 22 nt (CGGAACGGTGCGTA- GCACAATT) (EXIQON) [17] were used. The cDNA of *homer-1b* (NM001002496), *myf5* (NM131576) [19], *fmhc4* (NM001020485) [27], *smhc1* (NM001020507) [27] and *atp2a1* (NM001007029) [27] were used as probes.

### Western Blot

Analysis of total proteins was performed by Western blot on a 10% SDS-PAGE following the procedures described previously [28], except that the yolk was removed, and the antibodies of anti-Homer-1 (Santa Cruz) and α-tubulin (Sigma) were used at the dilution of 1∶400 and 1∶1000, respectively.

### Knockdown and Rescue Experiment

Antisense oligonucleotide morpholino (MO), *homer-1b*-MO, was designed specifically for translation inhibition of *homer-1b* (GGATCACCATTTCTTCATCCT-CCAT), which was complementary to nucleotides (nt) 131–155 of zebrafish *homer-1b* cDNA (NM001002496). Because Photo-MO can be induced at any desired stage to inhibit gene translation by UV light irradiation [29], [30], we also designed Photo-*homer-1b*-MO (GAGGATGAAPAAATGGTGATCC) which can release functional *homer-1b*-MO after UV exposure at 360 nm for 30 min. Additionally, *miR-3906-*MO (AAATCTGCATTCAAAATGCTTTTATCTACC) [17] was designed to knock down *miR-3906*. All MOs were prepared at 1 mM as a stock solution and were diluted to the desired concentration for microinjection, such as 8, 3, 2, or 1 ng per embryo. Capped mRNAs of *homer-1b* without the 3′ untranslated region (UTR), wobble mutated *homer-1b* without the 3′UTR, *homer-1b-*MO*-target-egfp* (in which the target sequence of *homer-1b-*MO was fused in frame with *egfp* cDNA), wobble mutated *homer-1b-target-egfp* (in which the mutated nucleotides of the target sequence of *homer-1b-*MO was fused in frame with *egfp* cDNA), and *egfp*, as well as pre*-miR3906* RNA, were synthesized according to the manufacturer’s protocol (Epicentre). The resultant mRNAs were diluted with distilled water to 172 ng/µl, 88 ng/µl and 44 ng/µl for wobble mutated *homer-1b* mRNA, 88 ng/µl for pre*-miR3906* RNA and 44 ng/µl for wobble mutated *homer-1b-target-egfp*, *homer-1b-*MO*-target-egfp,* and *egfp* mRNA. The total volume of approximately 2.3 nl was used for microinjection into one-cell embryos.

### Detection of the Distribution of Calcium Concentration within Zebrafish Embryos

A solution containing calcium green-1 (2 mM, Invitrogen) and dextran (tetramethylrhodamine 10000 MW, 0.5 µM, Invitrogen) was mixed with 8 ng of *miR-3906*-MO, 1.15 ng *miR-3906* dsR, 400 pg of *homer-1b* mRNA or 3 ng of *homer-1b-*MO and microinjected individually into one-cell zebrafish embryos. The fluorescent image of each embryo was captured by a Leica MZFLIII microscope equipped with a fluorescent system (Hg 100 W, with emission filters set at 488 and 583 nm) and a D3 camera (Nikon). The conditions for capturing the camera image were set at 1600 ISO and 2 sec exposure time for embryos at 24 hpf. We fixed a region consisting of 11 to 20 somites, except the artery and vein area, to calculate the emission reading of green and red for calcium green-1 and dextran, respectively, through ImageJ software. To rule out the injection volume difference of each embryo, the calcium green-1-light reading was divided by dextran red-light reading. To standardize the emission readings from embryos injected with *miR-3906* dsR, *miR-3906*-MO, *homer-1b* mRNA or *homer-1b*-MO, the reading obtained from WT embryos, which were only injected with calcium green-1 and dextran, was set as 100%. For the control group in each experiment, we took six WT embryos and set the averaged reading at 100% after deleting the highest and lowest reading values. In the experimental groups, we took an averaged reading from more than three readings each time. We repeated this experiment more than three times (n ≥3).

### Primary Cell Culture

The 24-hpf embryos derived from transgenic line *Tg(α-actin:RFP)* in which only the fast muscle cells are tagged by red fluorescence protein (RFP) [22] and microinjected separately with *miR-3906* dsR, *miR-3906*-MO, *homer-1b*-mRNA, *homer-1b*-MO, Photo-*homer-1b*-MO, pHsp-*miR-3906* or pHsp-*wob-homer-1b* were collected and washed three times with Hank’s saline. After washing, embryos were incubated in 0.5% bleach for 2–3 min and then washed three more times with Hank’s saline. The somites located at positions 11 through 20 of each embryo were dissected by forceps. The somites of 10 embryos were collected in a 1.5 ml Eppendorf tube and treated with 0.25% trypsin for 30 min at 28°C under a 5% CO_2_ incubator. Cells were pelleted by centrifugation and then resuspended in culture medium [31] in a volume of 600 µl. Cells cultured on dish at 28°C for eight hr under a 5% CO_2_ incubator were ready for [Ca^2+^]_i_ measurement. Before detecting [Ca^2+^]_i_ in the cells of embryos injected with plasmids pHsp-*miR-3906* and pHsp-*wob-homer-1b*, we heat-shocked these cultured cells in 37°C for four hrs.

### Quantification of [Ca^2+^]_i_ in the Fast Muscle Cells of Zebrafish Somites

Since we wanted to quantify the [Ca^2+^]_i_ in fast muscle-specific cells, we cultured cells from embryos of *Tg(α-actin:RFP)* whose fast muscle cells are tagged by RFP. After cells were specifically cultured for eight hr, they were incubated in the culture medium supplied with 5 µM Fura2-AM and 0.02% pluronic acid for 30 min in the dark at 28°C. To measure the [Ca^2+^]_i_ value in the fast muscle cells of zebrafish somites, we employed a fluorescence microscope (Leica DMI 4000) equipped with a 75 W Xenon lamp using 340 nm and 380 nm to excite Fura2-AM. The emission signal at 510 nm was received by CoolSNAP HQ2 CCD (Photometrics), and fluorescence intensities were analyzed by Metamorph software. To quantify [Ca^2+^]_i_, we followed the formula established by Grynkiewicz *et al*. [32]: [Ca^2+^]_i_ = *K_d_* × [(R–R_min_)/(R_max_–R)] × [F380_max_/F380_min_], where *K_d_* is the Fura-2 effective dissociation constant obtained from the Fura2-AM calcium imaging calibration kit (Molecular Probes). Somitic cells from *Tg(α-actin:RFP)* embryos without DNA/RNA injection served as an untreated control. When somitic cells were incubated for 4 hr with 772 µM of caffeine and 10 µM of 2-Aminoethoxydiphenyl borate (2-APB), which is a chemical that acts to block store-operated Ca ^2+^ entry, they served as positive and negative control, respectively.

### Rescue Experiments

Three types of rescue experiments were performed before the mRNA levels of *fmhc4*, *smhc1* and *atp2a1* were quantified at 24 hpf. First, we examined whether the expression defect caused by Photo-*homer-1b*-MO and pHsp-*miR-3906* could be rescued by coinjection of pHsp-*wob-homer-1b* and heat shock treatment at 20 hpf. The Photo-*homer-1b*-MO-injected embryos were exposed to UV at 20 hpf, and the pHsp-*miR-3906*-injected embryos were treated with heat shock. Second, we examined whether the expression defect caused by *miR-3906* dsR and *homer-1b-*MO could be rescued by soaking with caffeine (772 µM) between 20–24 hpf. Third, we examined whether the expression defect caused by *miR-3906*-MO and pHsp-*wob-homer-1b* could be rescued by incubation with 2-APB (10 µM). To induce *homer-1b* overexpression, the pHsp-*wob-homer-1b*-injected embryos were heat shocked at 37°C for 30 min when embryos developed at 20 hpf. After this treatment, both *miR-3906*-MO- and pHsp-*wob-homer-1b*-injected embryos were incubated in medium containing 2-APB.

### Quantitative RT–PCR (q-PCR)

Each experiment collected 100 embryos in 500 µl of Trizol Reagent (Invitrogen), which was stored at −80°C. Total RNA was isolated according to the manufacturer’s instructions. For quantitative PCR, first-strand cDNA was generated using 1 mg of total RNA. Both cDNA concentrations were adjusted to 200 ng/ml, and qPCR was performed using the 7900HT Fast Real-Time PCR System (Applied Biosystems, USA) according to the manufacturer’s instructions. Forward and reverse primers designed for cloning each gene by PCR were as follows, respectively: *homer-1b*, CCTTCATGGAAACTGCCTCAA and CACATCTACATCTCAGCGTC- TGC; *fmhc4*, GGAGGTTAAGGCTAAGAACGCACT and TCATGACGGGCTGAT- TGTACAG; *smhc1,* GTCAAGGATTCCCAAATGCAA and CCACAATGGCGAT- GTTCTCTTT; *atp2a1* (known as SERCA in mammals) [27], TCTGATCCCAG- TTCAGCTGCT and CATAACCTCCAATGGCCAGGT; *ef1a*, CTCCTCTTGGTCG- CTTTGCT and CCGATTTTCTTCTCAACGCTCT. Expression levels were determined by comparison with a standard curve from total RNA isolated from WT embryos.

### Phalloidin Staining and Transmission Electron Microscopy Examination

Phalloidin staining followed the procedures described previously [33], and Transmission Electron Microscopy followed the procedures described by Wang *et al.* [34] with some modifications. Embryos were fixed at 32 hpf with 2% paraformaldehyde and 2.5% glutaraldehyde in 0.1 M sodium cacodylate buffer, postfixed with 1% osmium tetroxide, dehydrated through a graded series of ethanol washes, and embedded in Spurr’s resin. Ultrathin (90 nm) sections were cut using an Ultracut E, stained with uranyl acetate followed by lead citrate (Reynolds), and the histological sections were examined under a Hitachi H-7650 transmission electron microscope (TEM) at 75 kV. To quantify the proportion of thick filaments (myosin heavy chain) versus thin filaments (sarcomeric actin) in the hexagonal arrangement shown on EM, we randomly selected five positions from the cross section of the EM image to count their numbers. The values were presented as the average of three independent trials.

## Results

### The Putative Target Genes of *miR-3906* by LAMP Assay and Microarray Analysis

The expression of *myf5,* a host gene of *miR-3906,* begins at 7.5 hpf in zebrafish somites and is predominant in the newly formed somites, gradually reducing its expression in mature somites [19]. We detected *miR-3906* in the front of mature trunk somites at 20 hpf [17], persisting up to eight days post-fertilization (8 dpf) in the muscle of zebrafish embryos (Fig. S1 in File S1). Therefore, we chose the stage during which zebrafish *myf5* significantly reduces in trunk muscles to study whether the persistence of *miR-3906* is able to functionally regulate its target genes in a manner that affects trunk muscle development.

We collected zebrafish embryos at 32 hpf and performed our LAMP assay, subsequently using the Zebrafish Whole Genome Microarray to analyze the RNA products pulled down by *miR-3906.* After normalization of the reading, we obtained 632 possible target genes of *miR-3906* and then selected the top 150 genes for further analysis with the NCBI and ZFIN databases. Only one third out of 150 genes could be defined, including *dkk3a*, which has already been reported as the target gene of *miR-3906* [17]. Based on the expression patterns of these 50 putative genes, we categorized them as genes belonging to the central nervous system, brain, eyes, muscles and other nonspecific regions (Fig. S2 in File S1). We focused on the following five muscle-specific genes for further study because we hypothesized that these genes would most likely be involved in the differentiation of muscle cells or modulation of muscle genes: *collagen type I alpha 2 (colla2), dnaJ homolog subfamily C member 10 (dnajc10), homer homolog 1b (homer-1b), sine oculis homeobox homolog 1a* (*six1a*) and *tRNA methyltransferase 2 homolog A* (*trmt 2a*).

### *miR-3906* Enables Silencing of Reporter Gene Fused by *homer-1b*-3'UTR Sequence

**Figure 1:**
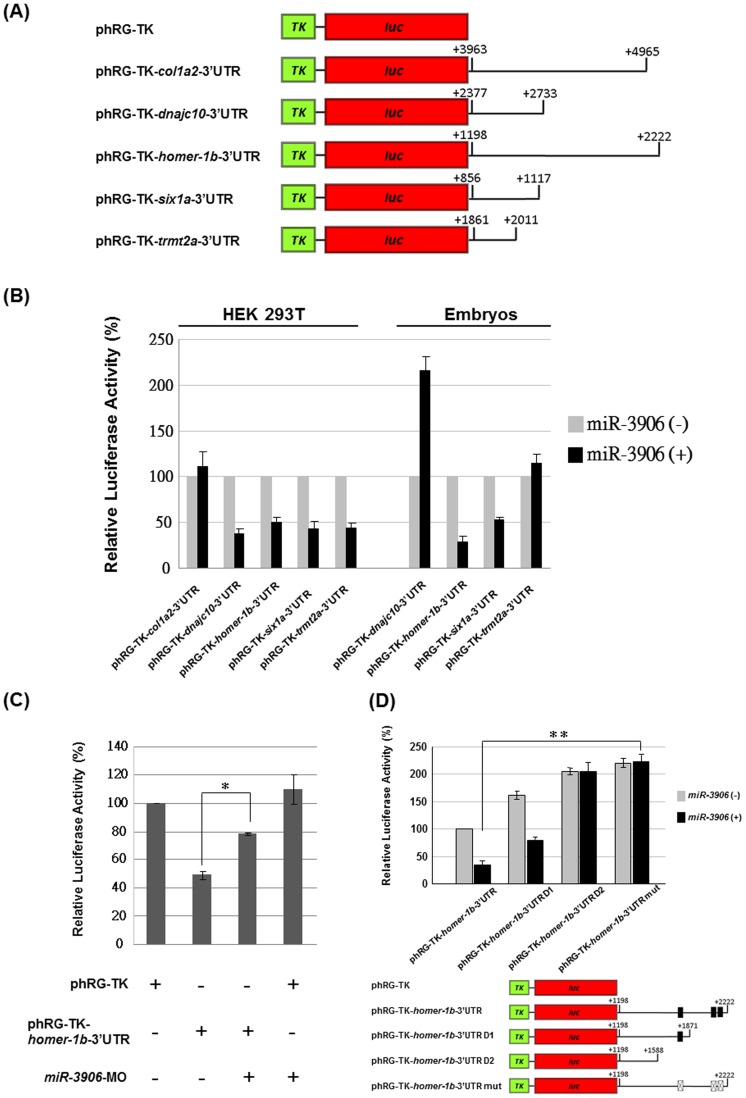
Luciferase (*luc*) activity assay of *miR-3906* co-injected with plasmid constructs containing 3′UTR segment of putative *miR-3906*-target genes. (**A**) Constructs for examining *luc* assay. The complete 3′UTR segments of *col1a2*, *dnajc10*, *homer-1b*, *six1a* and *trmt2a,* which are putative target genes of *miR-3906,* were ligated into the downstream of *luc* reporter gene contained in plasmid phRG-TK. (**B**) Plasmid pCMV-RFP-*miR-3906* [*miR-3906* (+)], a RFP reporter fused with pre-*miR-3906* and driven by CMV promoter, was co-transfected with pGL3-TK (internal control) and each examined construct, as indicated, into HEK 293T cells. Injection of pGL3-TK and each examined construct without containing pCMV-RFP-*miR-3906* [*miR-3906* (−)] served as a control group, and its *luc* activity was 100%. In zebrafish embryos, we co-injected synthetic pre-*miR-3906* RNA [miR-3906 (+)], pGL3-TK (internal control) and each examined construct in the experimental group. Injection of pGL3-TK and each examined construct without containing pre-*miR-3906* RNA [miR-3906 (−)] served as control group, and its *luc* activity was 100%. Data were presented as means±SD from three independent experiments (n = 3). (**C**) Injection of plasmid phRG-TK, in which *luc* expression was driven by thymidine kinase (TK), served as a control, and its *luc* activity was 100%. The relative *luc* expression level of each combination, as indicated, was examined. All data were presented as means±SD from three independent experiments (n = 3). (**D**) Various lengths of 3′UTR segment derived from *homer-1b* mRNA (from 1198 to 2222 nt) fused with *luc* reporter gene and driven by TK promoter were constructed as indicated. Plasmid alone or plasmid plus pre-*miR-3906* were individually injected in zebrafish embryos to perform *luc* assay. Data were presented as means±SD from three independent experiments (n = 3). Student's *t*-test determined significant differences between each group, and * indicates that the difference was significant at *P*<0.05. (black box: *miR-3906-*target sequences; cross filled box: *miR-3906*-target mutated sequences).

To examine whether *col1a2*, *dnajc10*, *homer-1b*, *six1a* and *trmt2a* are the target gene(s) of *miR-3906*, we constructed phRG-TK-*col1a2*-3′UTR, -*dnajc10*-3′UTR, -*homer-1b*-3′UTR, -*six1a*-3′UTR and -*trmt2a*-3′UTR (Fig. 1A). Compared with the *luc* activity from the control group in which pGL3-TK and each of above constructs were co-transfected, we normalized the *luc* reading from the experimental group in which pGL3-TK and each of examined constructs was co-transfected with pCMV-RFP-*miR-3906* individually into cell line HEK-293T. We found that *miR-3906* enabled reduction of *luc* expression through binding of the 3′UTR derived from *dnajc10*, *homer-1b*, *six1a* and *trmt2a*, compared to *luc* activity (100%) of the control group (Fig. 1B). We further performed *in vivo* screening of *dnajc10*, *homer-1b*, *six1a* and *trmt2a* in zebrafish embryos. After constructs of phRG-TK-*dnajc10*-3′UTR, -*homer-1b*-3′UTR, -*six1a*-3′UTR and -*trmt2a*-3′UTR were co-injected individually with pre-*miR-3906* RNA into one-cell embryos, we found that *miR-3906* enabled reduction of *luc* activity in embryos through binding of the 3′UTR derived only from *homer-1b* and *six1a* (Fig. 1B). Unexpectedly, when we compared the effects of *miR-3906* silencing on the 3′UTRs of *dnajc-10* and *trmt2a*, we noticed an inconsistency in the results between the HEK-293T cell line studied and the embryos. To explain this inconsistency, we speculated that (1) the special biological characteristics of various cell lines and (2) other unknown factor(s) that might be involved in the interaction between microRNAs and their target genes under a particular intracellular microenvironment. However, since investigating this interesting phenomenon is outside the scope of this paper, we decided to study *homer-1b* as its 3′UTRs could be greatly silenced by *miR-3906*, both *in vivo* and *in vitro*.

To confirm whether endogenous *miR-3906* in zebrafish embryos is able to inhibit reporter gene expression through binding *homer-1b*-3′UTR, we injected phRG-TK-*homer-1b*-3′UTR alone into embryos. Compared to the phRG-TK-injected control group, the *luc* activity of embryos injected with phRG-TK-*homer-1b*-3′UTR alone was greatly reduced (Fig. 1C), suggesting that endogenous *miR-3906* can functionally inhibit gene expression through 3′UTR derived from *homer-1b*. However, when *miR-3906* was knocked down by MO, which can specifically inhibit the generation of the mature form of *miR-3906*, and co-injected with phRG-TK-*homer-1b*-3′UTR, the *luc* expression level in zebrafish embryos increased (Fig. 1C). Furthermore, if *miR-3906*-MO was co-injected with phRG-TK, *luc* activity was close to that of embryos injected with phRG-TK alone (Fig. 1C), indicating that *miR-3906*-MO is not affected by TK activity. These lines of evidence suggest that the endogenous *miR-3906* in zebrafish embryos can specifically silence *luc* expression through the 3′UTR from *homer-1b* mRNA.

Next, in order to explore which lengths of *homer-1b*-3′UTR sequence might affect the inhibitory capabilities of *miR-3906*, several plasmids containing various deletion fragments from the full-length (+1198/+2222) of *homer-1b*-3′UTR fused with *luc* and driven by TK promoter were constructed, including D1 (+1198/+1871) and D2 (+1198/+1588) (Fig. 1D). Compared to the *luc* activity of embryos injected phRG-TK-*homer-1b*-3′UTR, which was set as 100%, the *luc* activities of embryos injected with phRG-TK-*homer-1b*-3′UTR D1 and phRG-TK-*homer-1b*-3′UTR D2 were 161.33±7.50% and 205.00±6.55%, respectively (Fig. 1D). However, the *luc* activities of embryos injected with pre-*miR-3906* RNA plus phRG-TK-*homer-1b*-3′UTR, phRG-TK-*homer-1b*-3′UTR D1 or phRG-TK-*homer-1b*-3′UTR D2 were 33.66±7.63%, 78.66±6.50% and 205.33±8.50%, respectively, suggesting that the full-length of 3′UTR sequence has the strongest *miR-3906-*mediated silencing ability. Furthermore, we used the *FindTar* [35], *Rna22* [36], and *RNAhybrid* [37] software to analyze this 3′UTR segment, and we found three putative binding sequences for *miR-3906* in *homer-1b*-3′UTR. We therefore mutated the nucleotides at these positions and constructed a plasmid phRG-TK-*homer-1b*-3′UTR mut (Fig. S3 in File S1). The *luc* activity of embryos injected with *miR-3906* plus phRG-TK-*homer-1b*-3′UTR mut was 222.33±12.89%, which exhibited a significant difference (p<0.05) from that of embryos injected with pre-*miR-3906* RNA plus phRG-TK-*homer-1b*-3′UTR (Fig. 1D). Taken together, we concluded that the *miR-3906* motif is capable of silencing the reporter gene through the binding of *homer-1b*-3′UTR.

### The Expressions of *homer-1b* and *miR-3906* are Co-localized in Trunk Muscle Cells

**Figure 2:**
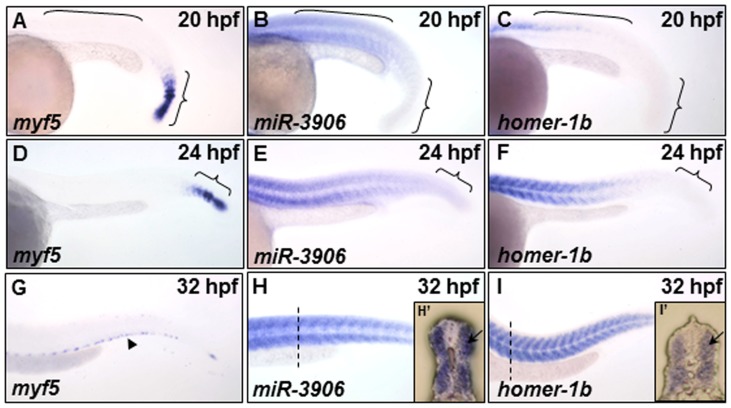
The expression patterns of *myf5*, *miR-3906* and *homer-1b* in the trunk somites of zebrafish embryos. (**A, D, G**) Using whole-mount *in situ* hybridization to detect the temporal and spatial expression patterns of *myf5*, (**B, E, H**) *miR-3906* and (**C, F, I**) *homer-1b* in zebrafish embryos at 20 hpf, 24 hpf and 32 hpf. At 20 hpf, (**A**) *myf5* was expressed in the pre-somitic mesoderm and newly formed somites (indicated by { ), but it was absent in mature somites (indicated by [); (**B**) *miR-3906* and (**C**) *homer-1b* were detected in mature somites. At 24 hpf, (**D**) *myf5* was detected in the pre-somitic mesoderm, while (**E**) *miR-3906* and (**F**) *homer-1b* gradually increased expression in mature somites. At 32 hpf, (**G**) *myf5* was expressed in the edge region of trunk muscles (arrowhead), but (**H**) *miR-3906* and (**I**) *homer-1b* were expressed in all trunk muscles. Cross section (dotted lines) showed that (**F’**) *miR-3906* and (**I’**) *homer-1b* were expressed in fast muscle (arrows).

We studied the spatiotemporal expressions of *homer-1b* in the muscle of zebrafish embryos (Fig. S4 in File S1), and we also studied the expression patterns of *miR-3906* and its host gene *myf5.* At 20 hpf, *myf5* was detected in the pre-somite mesoderms and some newly formed somites (Fig. 2A). At 20 hpf, *miR-3906* was expressed in the front of mature somites (Fig. 2B), while *homer-1b* was only starting to express in the front of mature somites (Fig. 2C). At 24 hpf, *myf5* expression was only detected in PSM (Fig. 2D). However, *miR-3906* was detected in most somites (Fig. 2E), and the expression of *homer-1b* increased in the front of mature somites (Fig. 2F). At 32 hpf, *myf5* was only detected at the edge of somites (Fig. 2G), while *miR-3906* and *homer-1b* were expressed in all trunk muscles (Figs. 2H and 2I). Using frozen sections, we found that *miR-3906* and *homer-1b* were expressed in the trunk fast muscle of zebrafish embryos (Figs. 2H’ and 2I’). Based on the significant reduction of host gene *myf5* in the trunk somites, these findings suggest that *miR-3906* and *homer-1b* were expressed in the fast twitch muscle tissue of somites in a co-localized manner.

### *miR-3906* Regulates the Expression Level of *homer-1b* mRNA in Zebrafish Embryos

**Figure 3:**
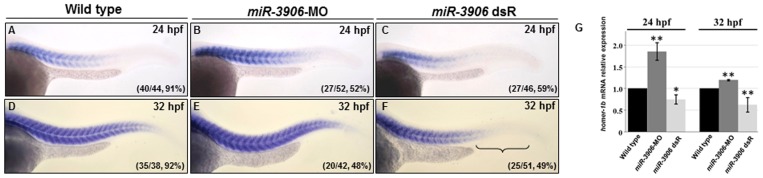
Change of *miR-3906* level affected the amount of *homer-1b* mRNA in zebrafish embryos. (**A**, **D**) Compared with WT, injection of *miR-3906*-MO increased the expression level of *homer-1b* mRNA in embryos at 24 hpf (**A** *vs*. **B**) and 32 hpf (**D** *vs*. **E**). Injection of *miR-3906* dsR (mature double-strand *miR-3906*) reduced the expression level of *homer-1b* mRNA in the front of trunk muscle and was absent in the tail region at 24 hpf (**A** *vs*. **C**) and 32-hpf (**D** *vs*. **F**). (**G**) The expression levels of *homer-1b* in WT embryos, *miR-3906*-MO-injected embryos and *miR-3906* dsR-injected embryos at 24 hpf and 32 hpf were quantified. Each one was carried out with 100 embryos, and triplicate experiments were performed (n = 3). The numbers shown in the lower-right corner of panels A–F indicate the number of phenotypes out of the total number of embryos examined. SigmaPlot software was used to perform Student’s *t*-test. *: indicates the difference at *P<0.05* level; **: indicates the difference at *P<0.01* level.

To confirm any correlation between the expression level of *miR-3906* and that of *homer-1b*, we injected either *miR-3906*-MO (8 ng) to inhibit the production of mature *miR-3906* or double-strand of mature *miR-3906* (*miR-3906* dsR) (1.15 ng) to overexpress *miR-3906* in zebrafish embryos before using WISH and q-PCR to detect the level of *homer-1b* mRNA. Compared with wild-type (WT) embryos at 24 and 32 hpf, the expression level of *homer-1b* mRNA increased in embryos injected *miR-3906*-MO (Figs. 3A and 3D *vs*. 3B and 3E). In contrast, the expression level of *homer-1b* mRNA in the embryos injected with *miR-3906* dsR decreased greatly in both trunk tail muscle and mature somites located in the front of trunk (Figs. 3C and 3F). Furthermore, using relative quantitative PCR, we compared the expression levels of *homer-1b* mRNAs among WT embryos, *miR-3906*-MO-injected embryos and *miR-3906* dsR-injected embryos, when the expression level of endogenous *homer-1b* mRNA in WT was set as 1. Results showed that the expression levels of *homer-1b* in the *miR-3906*-MO-injected and the *miR-3906* dsR-injected embryos at 24 hpf were 1.85±0.20 and 0.74±0.10, respectively; at 32 hpf, they were 1.19±0.01 and 0.62±0.16, respectively (Figs. 3G). The agreement of data between the q-PCR and WISH assays suggests that *miR-3906* negatively modulates the expression level of *homer-1b* mRNA.

### The Defective Phenotypes of *miR-3906* dsR-injected Embryos and *homer-1b*-MO- Injected Embryos are Similar

**Figure 4:**
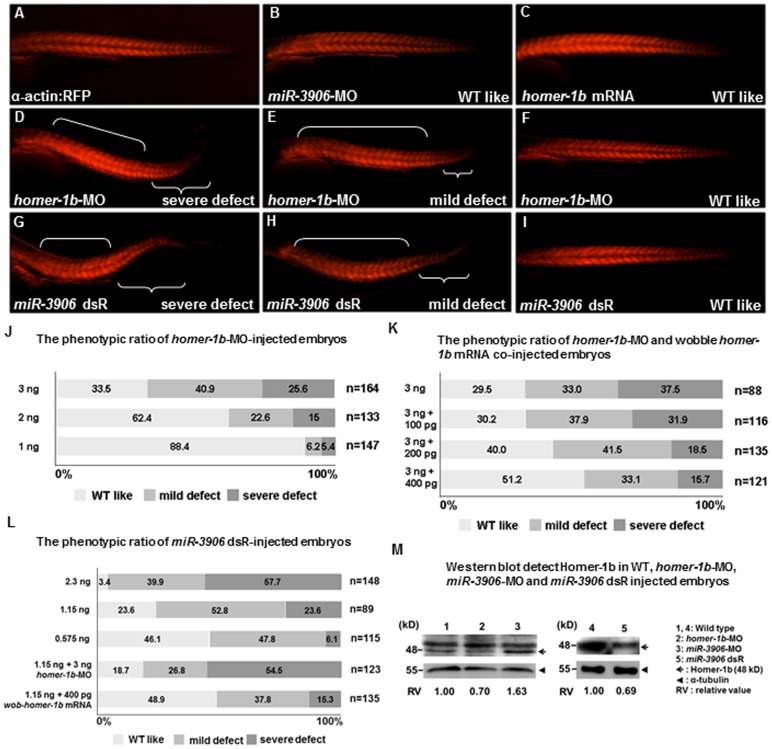
Defective phenotypes induced by injection of excessive *miR-3906* and *homer-1b*-MO were similar. (**A**–**I**) Embryos derived from transgenic line Tg (α-actin:RFP), in which red fluorescence protein (RFP) is expressed specifically in skeletal muscles, were injected with *miR-3906*-MO, *miR-3906* dsR (mature double- strand *miR-3906*), *homer-1b*-MO or *homer-1b* mRNA, as indicated. After injection, the development of trunk muscle was observed at 32 hpf. Compared with WT (**A**), the morphological trait of embryos injected with either *miR-3906*-MO (**B**) or *homer-1b* mRNA (**C**) was similar to that of WT. Embryos injected with *homer-1b*-MO (**D**–**F**) and exogenous *miR-3906* (*miR-3906* dsR) (**G**–**I**) displayed such defective phenotypes as body axis bending and trunk muscle shortening in tail. Based on the definition of three defective levels of muscle (see Materials and Methods), we calculated the defective percentages of embryos injected with *homer-1b*-MO alone (**J**) and *homer-1b*-MO plus wobble *homer-1b* mRNA (**K**). The occurrence percentages of severe and mild defects of embryos injected with 1.15 ng *miR-3906* dsR alone, 1.15 ng *miR-3906* dsR plus 3 ng *homer-1b*-MO and 1.15 ng *miR-3906* dsR plus 400 pg *homer-1b* mRNA were calculated (**L**). n: indicates the total number of embryos after three injections. (**M**) Western blot analysis of Homer-1b (48 kD, arrow) in WT (lanes 1 and 4), 3 ng *homer-1b*-MO-injected embryos (lane 2), 8 ng *miR-3906*-MO-injected embryos (lane 3) and 1.15 ng *miR-3906* dsR-injected embryos (lane 5). Detection of α-tubulin served as internal control (arrowhead). The relative intensity of Homer-1b protein among WT, *homer-1b*-MO, *miR-3906*-MO and *miR-3906* dsR was also indicated.

To confirm that *miR-3906* and *homer-1b* are involved in trunk muscle development through the same regulatory pathway, we injected *miR-3906*-MO, *miR-3906* dsR, *homer-1b*-MO and *homer-1b* mRNA individually into embryos derived from transgenic line *Tg(α-actin:RFP)*, in which the fast muscle is tagged by RFP. After injection, we were able to observe trunk muscle development *in vivo*. At 32 hpf, the morphological appearance of embryos injected with *miR-3906*-MO (8 ng) and *homer-1b* mRNA (400 pg) did not differ from that of WT (Figs. 4A, 4B and 4C). However, the swimming behavior of embryos injected with either *miR-3906*-MO or *homer-1b* mRNA was completely abnormal (Movie S1 in File S2). Injection of *homer-1b*-MO (Figs. 4D, 4E and 4F) or *miR-3906* dsR (Figs. 4G, 4H and 4I) exhibited the same defective phenotypes, such as body axis bending and tail muscle shortening.

Based on the trunk muscle defects observed, three levels of abnormality were categorized: (a) severe defect, i.e., significantly reduced muscle size after the 18th somite and bent body axis (Figs. 4D and 4G); (b) mild defect, i.e., muscle size starting to shorten after the 20th somite (Figs. 4E and 4H); and (c) WT-like, i.e., morphological appearance similar to that of WT (Figs. 4F and 4I). As shown in Figure 4J, we injected 3 ng, 2 ng and 1 ng of *homer-1b-*MO into WT zebrafish embryos and calculated the occurrence percentage of phenotypes suffering severe defect, mild defect and WT-like at 32 hpf. We found that the degree of phenotypic abnormality induced by *homer-1b*-MO was dose-dependent. Furthermore, when we co-injected 3 ng of *homer-1b*-MO with wobble *homer-1b* mRNA, which cannot be bound by *homer-1b*-MO (Fig. S5 in File S1), the percentage of injected embryos having severe defect was decreased, while the percentage of WT-like embryos was increased (Fig. 4K), indicating that the muscle defect was specifically caused by the loss of *homer-1b.* Next, as shown in Figure 4L, we injected 2.3 ng, 1.15 ng and 0.575 ng of *miR-3906* dsR into WT zebrafish embryos and calculated the occurrence percentage of phenotypes at 32 hpf. We found that the degree of phenotypic abnormality induced by *miR-3906* dsR was also dose-dependent. Furthermore, when we injected with 1.15 ng *miR-3906* dsR plus 3 ng *homer-1b*-MO, the percentages of injected embryos having severe defect, mild defect and WT-like were 54.5%, 26.8% and 18.7%, respectively. We also injected with 1.15 ng *miR-3906* dsR plus 400pg wobble *homer-1b* mRNA, and the percentages of injected embryos having severe defect, mild defect and WT-like were 15.3%, 37.8%, and 48.9%, respectively. Based on this evidence, we suggested that *miR-3906* and *homer-1b* are involved in trunk muscle development through the same regulatory pathways.

After WT embryos, *homer-1b*-MO-injected embryos, and *miR-3906*-MO- injected embryos were collected at 48 hpf, protein extracts were isolated, and Western blot analysis was performed using antibody against Homer-1b. We found that Homer-1b protein located at 48 kDa was present in the WT embryos (Fig. 4M, lanes 1, 4). However, Homer-1b protein was decreased in the embryos injected with *homer-1b*-MO (Fig. 4M, lane 2), indicating that the translation of *homer-1b* mRNA was specifically reduced by *homer-1b*-MO. In contrast, compared to WT, the protein level of Homer-1b was increased in the *miR-3906*-MO-injected embryos (Fig. 4M, lane 3) and reduced in the *miR-3906* dsR-injected embryos (Fig. 4M, lane 5), suggesting that *miR-3906* regulates Homer-1b protein expression and impacts the muscle development of zebrafish embryos.

### The Expression Levels of *miR-3906* and Homer-1b Affect Calcium Concentration in Fast Muscle Cells of Zebrafish Embryos

**Figure 5:**
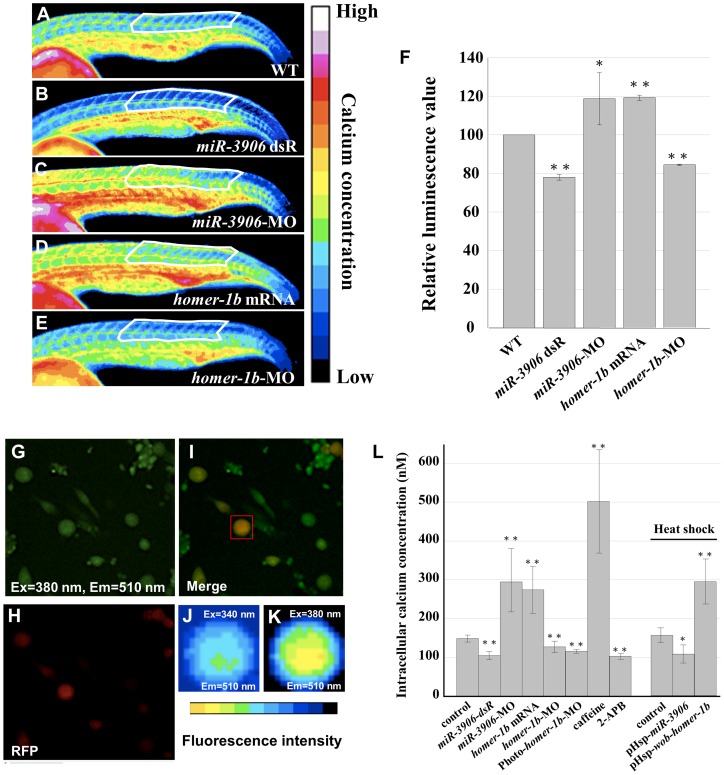
Injection of either *homer1b-*mRNA or *miR-3906-*MO increases the calcium concentration within muscle cells. (**A**) One-cell embryos were injected with buffer (A, as a control group WT), (**B**) *miR-3906* dsR (1.15 ng), (**C**) *miR-3906*-MO (8 ng), (**D**) *homer-1b* mRNA (400 pg) or (**E**) *homer-1b*-MO (3 ng) with calcium green-1 and tetramethylrhodamine (as an internal control). The gradient distribution of Ca^2+^ concentration within the muscle cells in somites was analyzed at 24 hpf by ImageJ software analysis. There were 16 colors of signal images displaying the different intracellular Ca^2+^ concentrations from low to high. We sketched a fixed region comprised of 11 to 20 trunk somites, as indicated, to calculate the readings from excitation emission (the region was indicated by white line). (**F**) After normalization, the reading from the WT group was set as 100, and the readings of *miR-3906* dsR-injected, *miR-3906*-MO-injected, *homer-1b-*mRNA-injected and *homer-1b-*MO-injected groups were compared. (**G**) The primary culture cells were stained with Fura2-AM, excited with 380 nm light, with emission at 510 nm. (**H**) The primary culture cells which displayed red fluorescence were the fast muscle cells. (**I**) The merge picture of F and G. Cells which had overlapping signals denoted in yellow were selected as detection samples (indicated by red box). (**J**) The image of fluorescence intensity shown on fast muscle cells that were excited by 340 nm, with emission at 510 nm. (**K**) The image of fluorescence intensity shown on fast muscle cells that were excited by 380 nm, with emission at 510 nm. (**L**) The value of [Ca^2+^]_i_ was calculated by following the formula established by Grynkiewicz *et al*. [31] (see Materials and Methods). The values of [Ca^2+^]_i_ were obtained from cells derived from WT, *miR-3906* dsR-injected, *miR-3906*-MO-injected, *homer-1b*-mRNA-injected, *homer-1b*-MO-injected, Photo-*homer-1b*-MO-injected, caffeine-soaked and 2-APB-soaked embryos, followed by heat-shocked WT, pHsp-*miR-3906*-injected and pHsp-*wob-homer-1b*-injected embryos. SigmaPlot software was used to perform Student’s *t*-test. *: indicates the difference at *P<0.05* level; **: indicates the difference at *P<0.01* level. Ex: excitation; Em: emission.

Homer-1b is a scaffold protein, not a transcription factor, in mammals [38]. It has been reported that Homer-1b binds ryanodine receptor to regulate calcium release from sarcoplasmic reticulum [21]. The calcium concentration in muscle cells is also known to affect muscle differentiation [39]. Therefore, we asked whether Homer-1b plays a role in fast-twitch muscle differentiation through modulating [Ca^2+^]_i_. To address this question, we injected calcium green-1, which can combine with intracellular calcium and release a green fluorescent signal [40], [41], in the one-cell stage of zebrafish embryos. Co-injection of tetramethylrhodamine, which is not affected by calcium ions [42], but appears as a red fluorescent signal, served as an internal control to monitor the volume of each injection. Based on the luminescent signal shown in Figure 5A, we observed the gradient distribution of calcium concentration in trunk from mature somites to newly forming somites in the WT embryos at 24 hpf. After the green signals were normalized, we found that the average reading score of green fluorescence in mature somites was higher than the calculated score for newly forming somites (data not shown). Thus, we selected somites from position 11 to 20, in which muscle cell differentiation is highly processed, for further study. The degree of green fluorescent signal indicated various concentrations of intracellular calcium in relation to the differentiation rate of muscle cells. Thus, in embryos injected with *miR-3906*-MO and *homer-1b* mRNA (Figs. 5C and 5D), results showed that the green fluorescence ratios were higher than WT. On the other hand, for embryos injected with *miR-3906* dsR and *homer-1b*-MO, the green fluorescence ratio was lower than that of WT (Figs. 5B and 5E), indicating that the green fluorescent intensity is reflective of [Ca^2+^]_i,_ which is controlled by the amount of Homer-1b protein in embryos. Additionally, when we quantified the relative luminescence value of signals among Figs. 5A–E, as shown in Fig. 5F, the reading from the WT group was set as 100. Compared to the WT control group, the readings of *miR-3906* dsR-injected, *miR-3906*-MO-injected, *homer-1b-*mRNA-injected and *homer-1b-*MO-injected groups were 78.00±1.45 (n = 3), 118.73±13.47 (n = 4), 119.25±1.35 (n = 3) and 84.45±0.33 (n = 3), respectively. We suggested that either reduction of *miR-3906* or enhancement of Homer-1b increased the amount of calcium in muscle cells. In other words, the decrease of Homer-1b resulted in lower [Ca^2+^]_i_ in muscle cells.

To accurately quantify [Ca^2+^]_i_ in fast muscle cells of somites, we chose to culture cells that were derived from 1) *Tg(α-actin:RFP)* embryos, since their fast muscle cells are tagged with RFP, and 2) somites only located at positions 11 to 20, since their fast muscle is newly synthesized at 24 hpf. After the fast muscle cells were identified by red fluorescence signal (Figs. 5G, 5H and 5I), they were excited at 340 nm and 380 nm separately, with emission at 510 nm (Figs. 5J and 5K). Based on the formula established by Grynkiewicz *et al*. [32] and *K_d_* of 420 nM, as obtained from the Fura2-AM calcium imaging calibration kit (Molecular Probes; see Materials and Methods), the calculated values of [Ca^2+^]_i_ of cells from embryos injected with *miR-3906* dsR, *miR-3906*-MO, *homer-1b*-mRNA, and *homer-1b*-MO were 104.78±10.4 nM (n = 21), 294.95±95.85 nM (n = 20), 274.11±60.81 nM (n = 28), and 127.62±14.73 nM (n = 22), respectively, whereas the calculated value of [Ca^2+^]_i_ of noninjected WT fast muscle cells was 148.75±9.20 nM (n = 25) (Fig. 5L). Meanwhile, the [Ca^2+^]_i_ values of cells from embryos incubated in caffeine, which served as positive control, and incubated in 2-APB, which served as negative control, were 501.92±132.99 nM (n = 24) and 102.97±8.17 nM (n = 22), respectively (Fig. 5L). Thus, compared to the noninjected WT control cells, the [Ca^2+^]_i_ values of cells from the *miR-3906*-MO-injected embryos and *homer-1b*-mRNA-injected embryos were increased 97.3% and 83.9%, respectively.

To exclude the possibility that the observed [Ca^2+^]_i_ change arising from the injection of *miR-3906-*MO and *homer-1b* mRNAs in embryos at the one-cell stage was the result of secondary effects, we further designed an experiment to induce the presence of MO and mRNA at the 20 hpf stage when endogenous *homer-1b* starts transcription (Fig. S4 in File S1). To accomplish this, we decided to employ Photo-morpholino technology whereby gene expression can be switched on and off by light. To first verify the efficiency and specificity of using Photo-*homer-1b*-MO, we introduced Photo-*homer-1b*-MO, together with *homer-1b*-MO-*target-egfp* mRNA, into zebrafish embryos. Results showed that reporter GFP was clearly expressed in embryos when not exposed to UV, whereas it was decreased dramatically when embryos were exposed to UV (Fig. S5 in File S1). This finding indicates that functional *homer-1b*-MO was released after UV exposure, resulting in downregulating the translation of the introduced *homer-1b*-MO-*target-egfp* mRNA. Thus, as a result of UV exposure, functional *homer-1b-*MO is released and then inhibits the translation of *homer-1b* mRNA, resulting in reducing Homer-1b protein. This evidence suggested that the use of Photo-*homer-1b*-MO to study late-stage embryo development is efficient and specific.

After we microinjected Photo-*homer-1b*-MO into one-cell embryos derived from transgenic line *Tg*(*α-actin:RFP*), embryos at 20 hpf were exposed to UV for 30 min. After UV exposure, the primary culture was carried out when embryos developed at 24 hpf. Fura2-AM staining showed that [Ca^2+^]_i_ of fast muscle of the Photo-*homer-1b*-MO-injected embryos was 116.03±4.96 nM (n = 31) (Fig. 5L), which was lower than that of noninjected control embryos at 148.75±9.20 nM (n = 25). We then microinjected plasmids pHsp-*miR-3906* and pHsp-*wob*-*homer-1b* into one-cell embryos of *Tg(α-actin:RFP)*, followed by heat shock at 37°C four hours prior to Fura2-AM staining. The [Ca^2+^]_i_ value of the noninjected, but heat shock-treated, control cells carrying no exogenous plasmids was 155.21±18.82 nM (n = 20) (Fig. 5L). However, the [Ca^2+^]_i_ values were 106.77±23.33 nM (n = 64) and 293.58±58.06 nM (n = 45) for cells carrying exogenous pHsp-*miR-3906* and pHsp-*wob*-*homer-1b,* respectively (Fig. 5L). Taken together, this evidence further confirmed that the change of expression level of either *miR-3906* or *homer-1b* mRNA resulted in a corresponding change of [Ca^2+^]_i_ in the fast muscle cells of zebrafish embryos.

### *miR-3906* and Homer-1b Affect Gene Expression in Fast Muscle Cells of Zebrafish Embryos by Changing [Ca^2+^]_i_

**Figure 6:**
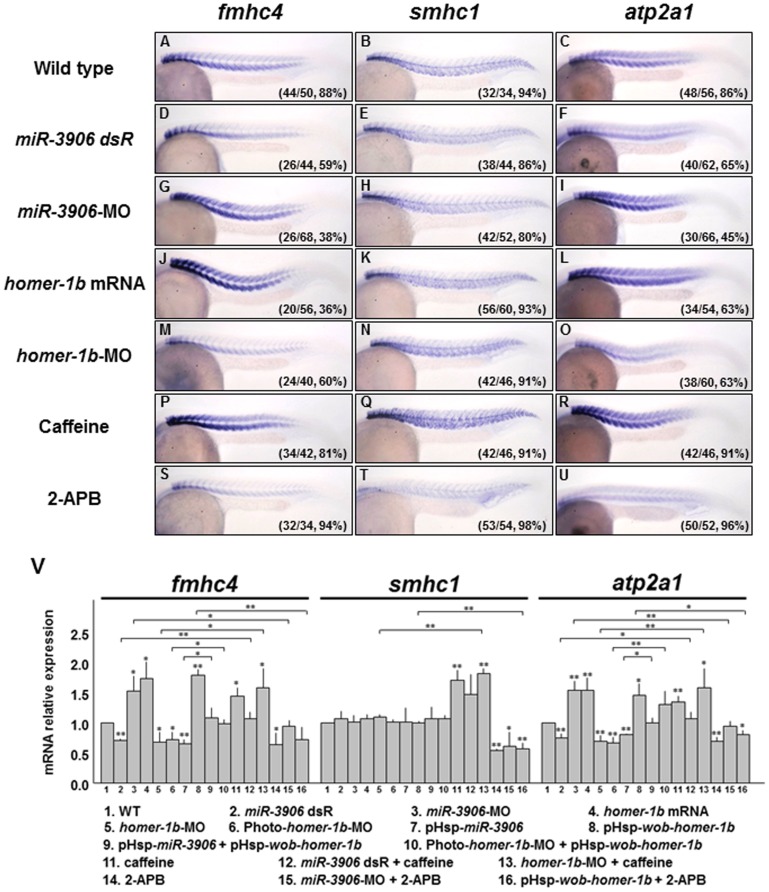
Injection of either *miR-3906-*MO or *homer-1b* mRNA affects fast muscle differentiation by regulating the calcium concentration in muscle cells. (**A–C**) Wild-type (WT) zebrafish embryos at 24 hpf and embryos injected with (**D–F**) *miR-3906* dsR (1.5 ng), (**G–I**) *miR-3906*-MO (8 ng), (**J–L**) *homer-1b* mRNA (400 pg), and (**M–O**) *homer-1b*-MO (3 ng) were collected, and the expressions of fast muscle type *fmhc4*, slow muscle type *smhc1*, and calcium-sensitive gene *atp2a1* were detected by WISH. (**P–R**) Embryos soaked with caffeine served as a positive control, indicating that [Ca^2+^]_i_ was increased. (**S–U**) Embryos soaked with 2-APB served as a negative control, indicating that [Ca^2+^]_i_ was reduced. Compared to WT (**A**), the expression of *fmhc4* in the embryos injected with *miR-3906*-MO (**G**), *homer-1b*-mRNA (**J**) and soaked in caffeine (**P**) was increased. The expression of *fmhc4* in the embryos injected with *miR-3906* dsR (**D**), *homer-1b*-MO (**M**) and soaked in 2-APB (**S**) was decreased. The expressions of *smhc1* in the embryos injected with *miR-3906* and *homer-1b* showed no difference (**B, E, H** and **K**), while *smhc1* expression was increased in embryos soaked in caffeine (**Q**) and reduced in embryos soaked in 2-APB (**T**). The expression patterns of *atp2a1* (**F, I, L, O, R** and **U**) in the examined embryos displayed a result similar to that of *fmhc4.* (**V**) The expression levels of the examined genes were quantified by q-PCR analysis after embryos were treated as indicated. Three rescue experiments were included: co-injection of pHsp-*wob-homer-1b* (lanes 9 and 10), soaking with caffeine (lanes 12 and 13) and incubating with 2-APB (lanes 15 and 16). SigmaPlot software was used to perform the Student’s *t*-test. *: indicates the difference at *P<0.05* level; **: indicates the difference at *P<0.01* level. The numbers shown in the lower-right corner of panels A–U indicate the number of phenotypes out of the total number of embryos examined.

To understand whether *miR-3906* and Homer-1b affected the gene expression in muscle cells of embryos by changing [Ca^2+^]_i_, we altered the expression levels of *miR-3906* and *homer-1b* and detected the expressions of the fast muscle-specific gene *fmhc4*, slow muscle-specific gene *smhc1,* and calcium-sensitive gene *atp2a1* in embryos at 24 hpf. Compared to the expression level of *fmhc4* in WT embryos (Fig. 6A), embryos injected with either *miR-3906*-MO or *homer-1b* mRNA showed increased *fmhc4* expression (Figs. 6G and 6J), as did embryos soaked in caffeine, which served as a control of increasing [Ca^2+^]_i_ (Fig. 6P) [43], [44]. However, *fmhc4* expression was decreased in embryos injected with either *miR-3906* dsR or *homer-1b*-MO (Figs. 6D and 6M), similar to embryos soaked in 2-APB, which served as a control of decreasing [Ca^2+^]_i_ (Fig. 6S) [45]. This evidence suggested that the gene expression in fast-twitch muscles is affected by [Ca^2+^]_i_. In contrast, the expression level of the slow muscle-specific gene *smhc1* exhibited little change in the embryos injected with either *miR-3906* or *homer-1b* (Figs. 6E, 6H, 6K and 6N), compared to that of wild-type embryos (Fig. 6B). We noticed that *smhc1* expression increased in the embryos soaked in caffeine (Fig. 6Q), whereas *smhc1* expression decreased in embryos soaked in 2-APB (Fig. 6T). These lines of evidence suggested that slow muscle gene expression is still affected by [Ca^2+^]_i_, but that *miR-3906* and *homer-1b* are not involved in gene expression of slow-twitch muscles. We noticed that the expression of the calcium-sensitive gene *atp2a1* was increased in caffeine-soaked embryos (Fig. 6R), while *atp2a1* expression was decreased in 2-APB-soaked embryos (Fig. 6U). In a parallel experiment, we found that *atp2a1* expression increased in the embryos injected with either *miR-3906*-MO or *homer-1b*-mRNA (Figs. 6I and 6L) because *miR-3906*-MO and *homer-1b*-mRNA enabled [Ca^2+^]_i_ to increase in embryos. However, *atp2a1* expression decreased in the embryos injected with either *miR-3906* dsR or *homer-1b*-MO (Figs. 6F and 6O) because *miR-3906* dsR and *homer-1b*-MO caused [Ca^2+^]_i_ to decrease in these embryos. In addition to analyzing the location and signal intensities of expressed genes using WISH, we also quantified the mRNA levels of expressed genes using q-PCR. As shown in Figure 6V, the mRNA levels of examined genes presented by q-PCR fundamentally agreed with those of data observed in WISH.

Furthermore, we wanted to clarify whether the downregulated expression of muscle genes in the presence of excessive *miR-3906* or absence of *homer-1b* could be rescued by conditionally adding exogenous wobble *homer-1b* at 20 hpf. To accomplish this, we exposed the Photo-*homer-1b*-MO-injected embryos under UV for 30 min at 20 hpf, and the expression of muscle genes, such as *fmhc4*,*smhc1* and *atp2a1*, was analyzed by qPCR at 24 hpf. Results showed that the expression of *smhc1* remained unchanged in the Photo-*homer-1b*-MO-injected embryos, whereas the expressions of *fmhc4* and *atp2a1* were relatively lower in the Photo-*homer-1b*-MO-injected embryos than those of the wild-type embryos (Fig. 6V): *fmhc4* was decreased by 27% (lanes 1 *vs*. 6 = 1∶0.73); *atp2a1* was decreased by 34% (lanes 1 *vs*. 6 = 1∶0.66). This evidence indicated that Photo-*homer-1b*-MO inhibits the translation of endogenous *homer-1b* mRNA, resulting in the reduction of Homer-1b, which, in turn, decreases the [Ca^2+^]_i_ within muscle cells.

Next, we injected Photo-*homer-1b*-MO together with plasmid pHsp-*wob-homer -1b*. It will be recalled that wobble *homer-1b* mRNA is a mutated form derived from *homer-1b* mRNA, in which the target sequences of *homer-1b*-MO are mutated, and the 3′UTR of *homer-1b* mRNA is not included, effectively eliminating targets for *miR-3906* and *homer-1b*-MO. We exposed these embryos under UV for 30 min and then performed heat shock treatment at 20 hpf. The gene expressions of *fmhc4* and *atp2a1* were analyzed by qPCR at 24 hpf. Compared to the 27% decrease of *fmhc4* expression in the Photo-*homer-1b*-MO-injected embryos, the decrease of *fmhc4* expression in the Photo-*homer-1b*-MO- plus plasmid pHsp-*wob-homer-1b*-injected embryos was only 2% (lanes 1 *vs*. 10 = 1∶0.98). Moreover, compared to the 34% decrease of *atp2a1* expression in the Photo*-homer-1b*-MO-injected embryos, the *atp2a1* expression in the Photo*-homer-1b*-MO- plus plasmid pHsp-*wob-homer-1b*- injected embryos was even increased up to 31% (lanes 1 *vs*. 10 = 1∶1.31). This line of evidence indicated that the overexpression of exogenous wobble-*homer-1b* mRNA induced at late stage, such as 20 hpf, rescues the decreases of *fmhc4* and *atp2a1* caused by Photo-*homer-1b*-MO (Fig. 6V).

Subsequently, we microinjected embryos with pHsp-*miR-3906* at the one-cell stage and carried out heat shock treatment for 30 min to overexpress *miR-3906* at 20 hpf. Similarly, the expression levels of muscle genes, such as *fmhc4*,*smhc1* and *atp2a1*, were then analyzed by qPCR at 24 hpf. Results showed that the expression of *smhc1* remained unchanged in the pHsp-*miR-3906*-injected embryos, whereas the expressions of *fmhc4* and *atp2a1* were relatively lower in the pHsp-*miR-3906*-injected embryos than those of the wild-type embryos (Fig. 6V): *fmhc4* was decreased by 32% (lanes 1 *vs*. 7 = 1∶0.68); *atp2a1* was decreased by 19% (lanes 1 *vs*. 7 = 1∶0.81). These results indicate that overexpression of *miR-3906* RNA after induction inhibits the translation of *homer-1b* mRNA, resulting in the decrease of [Ca^2+^]_i_.

We further microinjected plasmid pHsp-*miR-3906* together with plasmid pHsp-*wob-homer-1b* into zebrafish embryos at the one-cell stage, performed heat shock for 30 min at 20 hpf, and analyzed the gene expressions of *fmhc4* and *atp2a1* at 24 hpf. Compared to the 32% and 19% decreases of *fmhc4* and *atp2a1* expressions in the pHsp-*miR-3906*-injected embryos, respectively, the *fmhc4* and *atp2a1* expressions in the embryos injected with plasmids pHsp-*miR-3906* plus pHsp-*wob-homer-1b* were increased by 9% and 6%, respectively (Fig. 6V; *fmhc4*: lanes 1 *vs*. 9 = 1∶1.09; *atp2a1*, lanes 1 *vs*. 9 = 1∶1.06). This line of evidence indicated that the overexpression of exogenous wobble *homer-1b* after heat shock induction at late stage, such as 20 hpf, rescues the decreases of *fmhc4* and *atp2a1* expressions caused by *miR-3906*.

### The Phenotypes Caused by Manipulating either *miR-3906* or *homer-1b* Expression can be Rescued by Changing Calcium Concentration

We examined whether the abnormal expressions of muscle genes caused by manipulating the expression levels of *miR-3906* or *homer-1b* could be rescued by incubating embryos in medium containing a chemical which changes [Ca^2+^]_i._ First, at 20 hpf, the Photo-*homer-1b*-MO-injected embryos were exposed to UV, and the pHsp-*miR-3906*-injected embryos were treated with heat shock. The expressions of *fmhc4* and *atp2a1* were reduced in theses embryos (lanes 6 and 7, Fig. 6V). However, this defect could be rescued by co-injection of pHsp-*wob-homer-1b* and heat shock treatment at 20 hpf because the expressions of *fmhc4* and *atp2a1* reverted to normal levels (lane 6 *vs*. 10 and lanes 7 *vs*. 9, Fig. 6V).

Second, the expression levels of *fmhc4* and *atp2a1* in the *miR-3906* dsR (lane 2, Fig. 6V) or *homer-1b*-MO (lane 5, Fig. 6V) were lower than those of control WT embryos (lane 1, Fig. 6V) embryos. We examined whether the expression levels of muscle genes caused by excessive *miR-3906* or by knockdown of *homer-1b* could be rescued by soaking embryos in caffeine during 20–24 hpf. Results showed that the expression levels of *fmhc4* (lane 2 *vs*. 12, Fig. 6V) and *atp2a1* (lane 5 *vs*. 13, Fig. 6V) were greatly increased in the embryos injected with either *miR-3906* dsR or *homer-1b*-MO after soaking in caffeine, suggesting that caffeine increased [Ca^2+^]_i_ of fast muscles and restored the defective gene expressions induced by overexpression of *miR-3906* or by knockdown of *homer-1b*.

Third, we found that the expression levels of *fmhc4* and *atp2a1* in the embryos injected with *miR-3906*-MO and incubated with 2-APB (*miR-3906*-MO+2-APB) were both similar to their respective untreated control WT embryos (Fig. 6V, *fmhc4* section, lane 1 *vs*. 15 = 1∶0.94; *atp2a1* section, lane 1 *vs*. 15 = 1∶0.95). In contrast, when the *miR-3906*-MO-injected embryos were compared to the *miR-3906*-MO+2-APB-treated embryos, the mRNA expression levels of *fmhc4* and *atp2a1* were significantly decreased for the *miR-3906*-MO+2-APB-treated embryos (Fig. 6V, *fmhc4* section, lane 3 *vs*. 15 = 1.53∶0.94; *atp2a1* section, lane 3 *vs*. 15 = 1.54∶0.95). These results indicated that blocking calcium rise caused by *miR-3906*-MO could prevent changes of gene expression in fast muscle fibrils. Furthermore, we found that the expression levels of pHsp-*wob-homer-1b*+2-APB-treated embryos were slightly lower than those of untreated control WT (Fig. 6V, *fmhc4* section, lane 1 *vs*. 16 = 1∶0.72; *atp2a1* section, lane 1 *vs*. 16 = 1∶0.80). However, when the pHsp-*wob-homer-1b*-injected embryos were compared to the pHsp-*wob-homer-1b*+2-APB-treated embryos, the mRNA expression levels of *fmhc4* and *atp2a1* were significantly decreased for the pHsp-*wob-homer-1b*+2-APB-treated embryos (Fig. 6V, *fmhc4* section, lane 8 *vs*. 16 = 1.79∶0.72; *atp2a1* section, lane 8 *vs*. 16 = 1.46∶0.80). These results also indicated that blocking calcium rise caused by *homer-1b* could prevent changes of gene expression in fast muscle fibrils.

### *miR-3906* Inhibits Homer-1b Expression to Avoid Excessive Calcium Concentration in Muscle Cells

**Figure 7:**
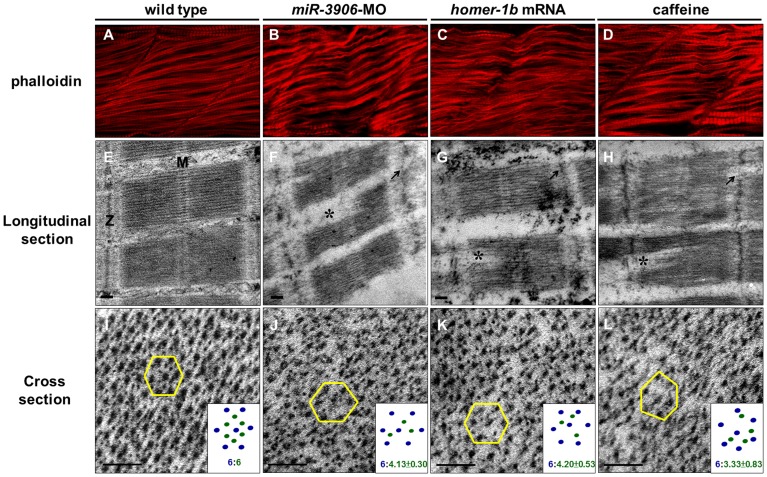
Either inhibition of *miR-3906* or overexpression of *homer-1b* disrupts sarcomeric actin organization. Using phalloidin stain, compared to (**A**) WT embryos, (**B**) *miR-3906-*MO-injected embryos, (**C**) *homer-1b-*mRNA-injected embryos and (**D**) caffeine-soaked embryos exhibited a bending and disruption of sarcomeric actin organization in muscle cells. In longitudinal section, compared to (**E**) WT control embryos, (**F**) the Z-disc structure displaying in *miR-3906-*MO-injected embryos, (**G**) *homer-1b-*mRNA-injected embryos and (**H**) caffeine-soaked embryos was chaotic (indicated by arrows), and the full-length sarcomeres in muscle fibers were interrupted (indicated by asterisks) and could not be seen completely in a full sarcomere line. In cross section, compared to (**I**) WT control embryos, the relative position and proportion between myosin heavy chain and actin filament among the (**J**) *miR-3906-*MO-injected embryos, (**K**) *homer-1b-*mRNA-injected embryos and (**L**) caffeine-soaked embryos were chaotic and extremely irregular. For each sample, we randomly selected five positions to identify their hexagonal arrangements and calculated the ratio between the thick and thin filaments (n = 3). Results were presented at the bottom right of each picture (**I–L**). M:M line;Z:Z-disc;scale bar:0.1 µm.

Although no significant deficiency was observed in external muscle morphology between wild-type embryos and embryos injected with *miR-3906*-MO and *homer-1b*-mRNA (Figs. 4B and 4C), the swimming behavior of larvae derived from either *miR-3906*-MO-injected embryos or *homer-1b*-mRNA-injected embryos was abnormal (Movie S1 in File S2). This fact prompted us to study whether the high concentration of calcium impacts muscle structure. We used phalloidin staining and confocal microscopy to examine fast muscle sarcomeric actin arrangement in fast muscle fibrils of embryos at 32 hpf. Sarcomeric actin arrangement showed fast muscle fibers in WT embryos as oriented in the same direction with clear dye-sarcomere boundaries (Fig. 7A). In comparison, the sarcomeric actin arrangement was bent with indistinct dye-sarcomere boundaries in the *miR-3906*-MO-injected embryos (Fig. 7B), *homer-1b*-mRNA-injected embryos (Fig. 7C), pHsp-*homer-1b*-injected (Fig. S6 in File S1) and caffeine-soaked embryos (from 20 to 32 hpf) (Fig. 7D). We further applied transmission electron microscopy to observe the sarcomeric ultrastructure in muscle cells. No defect of the sarcomeric ultrastructure could be seen in WT embryos (Fig. 7E), whereas the sarcomeric ultrastructure was disordered in the embryos injected with *miR-3906*-MO (Fig. 7F), injected with *homer-1b*-mRNA (Fig. 7G), or soaked in caffeine (Fig. 7H), because of the bent muscle fibers. In addition, the boundary structure between sarcomeres, such as actin filament bound by Z disk, could not be clearly defined. In WT embryos, a regular hexagonal arrangement of thick filament intercalating with thin filament could be seen in cross sections at a ratio of thick: thin = 6∶6 (Fig. 7I). In comparison, the relative location between the myosin heavy chain and actin filament was seriously disordered in *miR-3906*-MO-injected embryos (thick: thin = 6∶4.13±0.30) (Fig. 7J), *homer-1b*-mRNA-injected embryos (thick: thin = 6∶4.20±0.53) (Fig. 7K), and caffeine-soaked embryos (thick: thin = 6∶3.33±0.83) (Fig. 7L), indicating that the proportional arrangement between myosin heavy chain and actin filament was chaotic in the *homer-1b*-overexpressed embryos. This evidence suggested that higher calcium concentration in muscle cells causes loss of organization in sarcomeric actin arrangement, which, in turn, causes defective muscle development.

### The Upstream *cis-*element is Capable of Driving the Transcription of *miR-3906* When the Host Gene *myf5* Transcription is Absent

**Figure 8:**
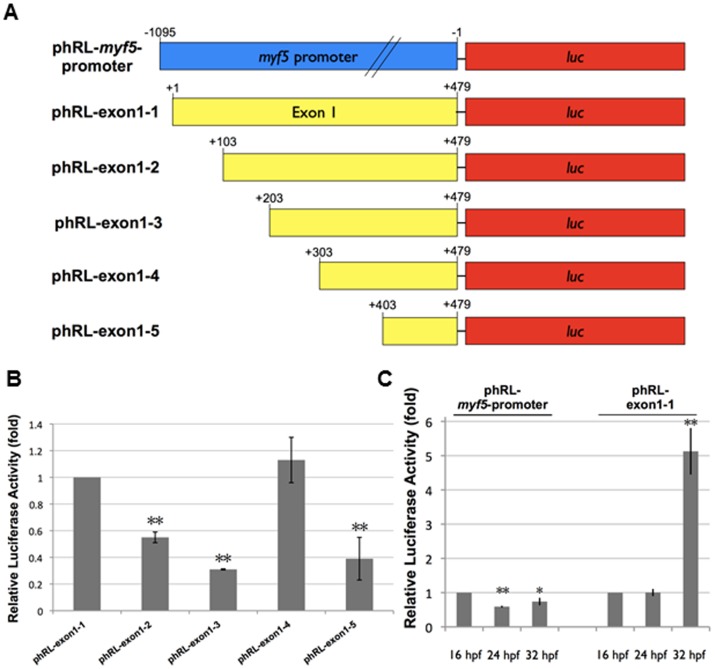
The proximal upstream *cis*-elements of *miR-3906* are capable of controlling the transcription of *miR-3906*. (**A**) The schematic diagrams of plasmid constructs having a 1.1 kb fragment upstream of *myf5* gene and having various deletions of exon 1 of *myf5* which are proximal upstream regulatory segments of *miR-3906.* (**B**) Luciferase activity assay in zebrafish embryos at 24 hpf. Plasmids phRL-exon1-1, phRL-exon1-2, phRL-exon1-3, phRL-exon1-4 and phRL-exon1-5 were co-injected individually with pGL3-TK, an internal control, into one-cell embryos. The *luc* expression of embryos injected with plasmid phRL-exon1-1 served as 1. The relative *luc* expression level of each combination was examined. (**C**) Luciferase activity assay in zebrafish embryos at 16, 24 and 32 hpf. Plasmids phRL-*myf5*-promoter and phRL-exon1-1 were co-injected individually with pGL3-TK, an internal control, into one-cell embryos. The *luc* expression of each plasmid at 16 hpf served as 1. The relative *luc* expression levels of phRL-*myf5*-promoter and phRL-exon1-1 at 24 and 32 hpf were examined. Data were presented as means±SD from three independent experiments (n = 3). Student's *t*-test was used to calculate the differences among data. *: indicates significant difference at *P<0.05*. **: indicates significant difference at *P<0.01*.

To prove whether the upstream *cis-*element is capable of driving the transcription of *miR-3906*, we constructed plasmids in which *luc* reporter was driven by various lengths of upstream segment located from +1 to +479 (+1/+479) (Fig. 8A). After injection, we found that the luciferase activities of embryos injected with those plasmids missing the +203/+302 segment (phRL-exon1-3) and the +303/+402 segment (phRL-exon1-4) increased and decreased greatly, compared to those of embryos injected with plasmids containing phRL-exon1-3 and phRL-exon1-4, respectively (Fig. 8B). In addition, the proximal upstream sequence of *miR-3906* was capable of driving reporter gene activity, but with a greater driving strength at later developmental stages, such as 32 hpf, when the expression of host gene *myf5* becomes weaker in trunk muscle than that observed at early developmental stages, such as 16 hpf, when *myf5* expression is strong in trunk (Fig. 8C). This evidence suggested that the upstream elements of *miR-3906* are capable of controlling the transcription of *miR-3906*, including segments such as +203/+302 and +303/+402, which are responsible for inhibition and activation of *miR-3906* transcription, respectively. Therefore, we hypothesize that *miR-3906* might be transcribed from the activation of segment +303/+402, when host gene *myf5* apparently disappears from trunk muscle cells at later developmental stages, thereby reducing Homer-1b protein level in zebrafish embryos.

## Discussion

### Regulation of Intracellular Calcium Concentration by Homer-1b Affects Muscle Development

Ca^2+^ channels, such as IP3R, RyR and TRPC, are expressed in skeletal muscle cells, and Homer proteins can bind them individually [46]–[48]. IP3R and RyR channels are located at SR membrane and are responsible for releasing Ca^2+^ from SR into cytoplasm. After differentiation of muscle cells, Stiber *et al.* [49] found that Homer proteins increase their expression levels greatly. For example, Homer-1 and Homer-2b are expressed to the degree required to release Ca^2+^ through the RyR channel to increase intracellular [Ca^2+^]. When the RyR channel is activated, nuclear factor of activated T cells (NFAT) enters the nucleus to turn on the expression of downstream muscle genes. Thus, Homer-1 protein not only has the ability to regulate the activation of the RyR channel, but also affects Ca^2+^-dependent signaling transduction during muscle cell differentiation.

In this *in vivo* study of zebrafish embryos, we demonstrated that Homer-1b regulates intracellular [Ca^2+^]. Specifically, we found that overexpression of *homer-1b* mRNA increases intracellular [Ca^2+^], while injection of *homer-1b*-MO decreases [Ca^2+^]_i_ in muscle cells (Fig. 5). Meanwhile, since a) *homer-1b* mRNA was expressed at higher level and b) [Ca^2+^]_i_ was increased in muscle cells of the *miR-3906-*MO-injected embryos, we concluded that *miR-3906* affects [Ca^2+^]_i_ in muscle cells by decreasing the translation of *homer-1b* mRNA (Fig. 5). We also observed that the release of [Ca^2+^]_i_ was enhanced in the embryos soaked in caffeine, resulting in the increased expression of such fast muscle-specific genes as *fmhc1* and *atp2a1*. In contrast, the release of [Ca^2+^]_i_ was reduced in the embryos soaked in 2-ABP, resulting in the decreased expressions of *fmhc1* and *atp2a1* (Fig. 6), suggesting that the fast muscle gene expression of zebrafish is controlled by [Ca^2+^]_i_. Additionally, we demonstrated that the phenotypes caused by manipulating either *miR-3906* or *homer-1b* expression can be rescued by changing calcium concentration. Therefore, we concluded that the *miR-3906* or *homer-1b* system affects gene expression in the fast muscle fibrils, such as *fmhc4*and *atp2a1*, as a result of change in calcium influx and not through direct influence on the promoter activity of genes or the stability of mRNA. Thus, *miR-3906* is capable of fine tuning [Ca^2+^]_i_ in muscle cells through targeting *homer-1b* mRNA to decrease the protein level of Homer-1b, leading to decreased expression of fast muscle-specific genes (Fig. 6). On the other hand, irrespective of which method was employed to increase [Ca^2+^]_i_, i.e., by injection of *miR-3906*-MO or *homer-1b* mRNA or by soaking in caffeine, the expressions of *fmhc1* and *atp2a1* did not exhibit a significant difference in terms of somite number, compared to the wild-type embryos. Therefore, we believe that the control of muscle cell differentiation still requires the involvement of MRFs, such as *myod* and *myogenin*, while the increase of [Ca^2+^]_i_ serves to provide an intracellular condition that favors the phosphorylation of MEF2 and NFAT in muscle cells to activate the expression of downstream genes.

### Homeostasis of Intracellular Calcium Concentration is Required in Order to Maintain Normal Muscle Cell Development and Function

Morphological defects in muscle development were not found in either *miR-3906*-MO-injected embryos or *homer-1b-*mRNA-injected embryos, as demonstrated in this study. We did, however, observe a defect in swimming ability, including short-distance escape swimming and abnormal rotation, in the embryos injected with either *miR-3906*-MO or *homer-1b* mRNA. When we examined muscle structure under confocal microscopy, we found that both *miR-3906*-MO-injected embryos and *homer-1b*-mRNA-injected embryos exhibited a bent arrangement of muscle fibers, indistinct sarcomere boundaries, and lost organization of sarcomeric actin. These structural defects of muscle cells were also observed in the caffeine-soaked embryos. Furthermore, when we used electron microscopy to observe the ultrastructure of muscle cells, we found irregular arrangements in three kinds of embryonic sarcomere, vague Z line, and chaotic position between myosin heavy chain and actin filament (Fig. 7). This line of evidence suggested that excessive [Ca^2+^]_i_ induced by injection of either *miR-3906*-MO or *homer-1b* mRNA causes ultrastructural defects in muscle cells. Interestingly, the defects found in zebrafish are similar to those found in mice, as reported by Stiber *et al.* [50], who demonstrated that *homer-1*-knockout mice displayed smaller myofibers and weaker muscle contraction. This myopathic phenomenon is conclusive for increased intracellular calcium concentration. Additionally, Millay *et al.* [51] reported that overexpression of transient receptor potential canonical 3 (TRPC3) causes an influx of intracellular calcium, leading to muscular dystrophy, which is similar to the dystrophin-glycoprotein complex (DGC)-deficient model phenotype. They also found that muscle gene expression was similar to that of the DGC-deficient dystrophic disease model. In this study, we also found a similarity between muscle atrophy in the trunk and tail of embryos injected with *homer-1b*-MO (Fig. 4) and the abnormal pattern of shortened somites in *camk2b*-MO-injected embryos [52]. This evidence suggested that loss of Ca^2+^-dependent signaling leads to more serious defects in muscle development. Therefore, either higher [Ca^2+^]_i_ or lower [Ca^2+^]_i_ causes muscle development defects, indicating homeostasis of [Ca^2+^]_i_ in muscle cells is critical for normal muscle development and function at the differentiation stage. These findings lead to the conclusion that *miR-3906* functions to fine tune the protein level of Homer-1b, which, in turn, affects [Ca^2+^]_i_ homeostasis in muscle cells to control fast muscle differentiation rate and normal muscle structure and function.

### Similar to *miR-206*, Different Target Genes of *miR-3906* Play Unique Roles at each Stage of Embryonic Development

In responding to different developmental stages, miRNA plays a unique role in the dynamic process of embryo development. Since, for example, it has been shown that *miR-206* has different target genes at different stages of mouse embryonic development [4], we hypothesized that *miR-3906* might also target specific genes affecting muscle development during embryonic development.

To illustrate this phenomenon, *miR-206* has been shown to impact the function of transcription factor Pax3 in muscle cells. Specifically, in early muscle development, the dermomyotome cells migrate to the limb bud and serve as precursor cells to form the limb muscles. Pax3 in dermomyotome cells modulates specialization and proliferation in muscle cells through its control of Myf5 and Myod [53], [54]. Goljanek-Whysall *et al.* [55] found that Pax3 is highly expressed in mice in the muscle cells of forelimbs and hindlimbs at HH24, whereas Pax3 is greatly decreased at HH28. In contrast, while *miR-206* expression increases in limbs at HH24, it decreases at HH28. Thus, it appears that the expression of both *pax3* and *miR-206* in mouse limbs is dynamic and stage-dependent. Additionally, knockdown of *miR-206* increases both the mRNA level and protein level of Pax3, suggesting that the expression of *pax3* is negatively correlated with that of *miR-206* and that *miR-206* can control *pax3* gene expression at the posttranscriptional level. Therefore, silencing of *pax3* by *miR-206* at the early somatic muscle developmental stage causes decreased specification and proliferation of muscle cells which, in turn, favors further differentiation of muscle cells. It has also been shown that *miR206* is involved in TGF-β signaling which inhibits muscle cells from further differentiation. In this case, histone deacetylase 4 (HDAC4) is one of the downstream effectors of TGF-β. When muscle cells begin to differentiate, *miR-206* silences HDAC4, allowing cells to differentiate and produce muscle regulatory protein MEF2 and muscle structure protein MHC [56]. At the same time, however, *miR-206* can also silence the expression of tissue inhibitor of metalloproteinase 3 (TIMP3), which inhibits TNFαfrom downregulating the process of differentiation. Since TIMP3 is decreased by *miR-206*, the release of TNFαis increased, which results in enhancing the phosphorylation of p38 and expression of the muscle structural proteins Myogenin and MHCs. Under these conditions, the muscle cells finally proceed to differentiation [57].

Interestingly, in this zebrafish study, we found some of these same characteristics in *miR-3906*. First, we observed that *miR-3906* is detectable from 16 hpf [17]. We could also detect the presence of *miR-3906* in the trunk muscles, even as late as 8 dpf. At 16 hpf, *miR-3906* is significantly increased in mature somites. As a result, the expression of *dkk3a,* a target gene of *miR-3906,* is inhibited in mature somites. Since *dkk3a* is decreased, the promoter activity of *myf5* is immobilized. This then results in the significant reduction of *myf5* expression in mature somites such that the expression of *myf5* is almost undetectable in somites after 24 hpf. Taken together, this evidence suggested that *miR-3906* reduces the specification of muscle cells, but facilitates the process of differentiation. Interestingly, during muscle cell differentiation after 24 hpf, *homer-1b*, another target gene of *miR-3906*, functions to increase the [Ca^2+^]_i_ of muscle cells to facilitate fast muscle gene expression. At this stage, *miR-3906* reduces the level of Homer-1b protein to maintain [Ca^2+^]_i_ homeostasis. Therefore, similar to *miR-206*, which plays different functional roles in embryogenesis through its target genes, we concluded that *miR-3906* inhibits the expression of either *dkk3a* or *homer-1b* at different developmental stages to ensure normal muscle development during myogenesis in embryos by facilitating specification or differentiation, respectively.

### *miR-3906* Fine Tunes *homer-1b* Expression and Affects Trunk Muscle, even in the Absence of Host Gene *myf5* Transcription

Some intronic miRNAs are reported to be expressed at developmental stages absent transcription of host genes [58]–[60]. This phenomenon supports observations presented in this study. That is, although *miR-3906* is derived from the *myf5* transcripts in somites, the expression of *myf5* is gradually reduced to an undetectable level in mature trunk somites after 20 hpf. Nonetheless, *miR-3906* is continuously detectable in the front of mature trunk somites at 20 hpf, even persisting up to 8 dpf. Additionally, Mi *et al.* [61] demonstrated that *has-miR-128b* is overexpressed in blood cells of acute lymphoblastic leukemia patients when the promoter of its host gene ARPP21 is modified by hypomethylation and the promoter activity is decreased, indicating that the expression of *has-miR-128b* is controlled by its own promoter. In this study, we supported the hypothesis raised by the above reports because we proved that intronic *miR-3906* is controlled by its own promoter at later developmental stages, thus providing an alternative explanation for the transcriptional decoupling between *miR-3906* and its host gene *myf5.*

### Conclusion

**Figure 9:**
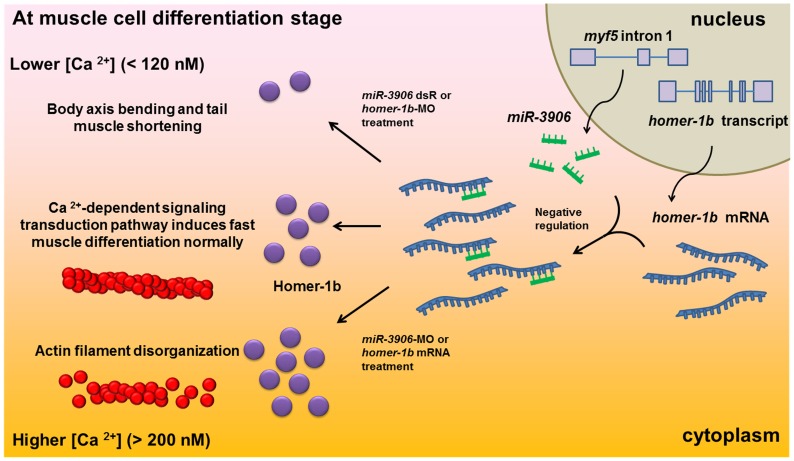
Schematic model illustrating how *miR-3906* controls [Ca^2+^]_i_ homeostasis in fast muscle cells during differentiation. Overexpression or knockdown of either *miR-3096* or *homer-1b* causes an imbalance between *miR-3096* and *homer-1b,* resulting in disturbing [Ca^2+^]_i_ homeostasis in the fast muscle cells during differentiation. At this developmental stage, *miR-3906*, which is transcribed from its own promoter, decreases [Ca^2+^]_i_ through inhibiting the translation of *homer-1b* mRNA and causing the reduction of Homer-1b protein. This fine-tuning of the protein amount of Homer-1b by *miR-3096* allows *homer-1b* to be present at a normal level in order to maintain [Ca^2+^]_i_ homeostasis, thus ensuring normal fast muscle differentiation.

Based on the above evidence, as depicted in Figure 9, we concluded that normal muscle cell development during differentiation is characterized by [Ca^2+^]_i_ homeostasis controlled by the negative regulation of Homer-1b concentration through *miR-3906* in fast muscle fibrils, allowing Ca^2+^-dependent signaling transduction to induce the expression of fast muscle genes. However, when either *miR-3906* or *homer-1b* mRNA expression is downregulated or overexpressed, a state of [Ca^2+^]_i_ imbalance occurs, resulting in abnormal zebrafish embryonic development. Briefly, when excessive *miR-3906* RNA is introduced or *homer-1b*-specific MO is injected to knock down *homer-1b*, the decrease in [Ca^2+^]_i_, e.g., below 120 nM, in muscle cells results in such severe consequences as body bending and tail shortening defects. On the other hand, when *miR3906*-specific MO is injected to knock down *miR-3906* or excessive *homer-1b* mRNA is introduced, the increase in [Ca^2+^]_i_, e.g., over 200 nM, in muscle cells results in such developmental defects as sarcomeric actin disorganization and abnormal swimming behavior. Thus, *miR-3906*, by its fine tuning of *homer-1b* expression, functions delicately in muscle differentiation at the later developmental stage.

## Supporting Information

File S1 **File containing Figures S1–S6.** **Figure S1.** *miR-3906* persisted in the trunk muscle of zebrafish embryos at later developmental stages. (**A**, **D**) Using WISH to detect the expression of *miR-3906*, (**B**, **E**) *miR-206* and (**C**, **F**) *myf5* intron1 control-22nt of zebrafish embryos at 48 hpf and 8 dpf. (**A**) *miR-3906* was detected both at 48 hpf and (**D**) 8 dpf in the trunk muscles of zebrafish embryos. (**B**, **E**) Detecting *miR-206* expression served as a positive control, (**C**, **F**) while detecting the expression of *myf5* intron1 control-22nt served as negative control. **Figure S2.** The putative target genes of *miR-3906* using LAMP assay and microarray analysis. LAMP assay was performed for the cell extracts from zebrafish embryos at 32 hpf, and the possible target-RNAs of *miR-3906* were analyzed by Zebrafish Whole Genome Microarray (Aligent). After standardization, 632 possible target genes of *miR-3906* were predicted. Among them, 150 genes with higher binding capacity were selected and the NCBI’s ZFIN database used to define these plausible genes. Only 50 out of 150 genes had a complete gene sequence and were defined, including, for example, 4 genes of brain, 3 of the central nervous system (CNS), 1 of lens, 12 of muscles and 30 others from nonspecific regions. NA: not available. **Figure S3.** The mutated sequences of *miR-3906* binding sites at the 3′UTR of *homer-1b* mRNA. (**A**) Three *miR-3906* binding sequences at the 3′UTR of *homer-1b* mRNA were predicted at positions 1, 2 and 3. (**B**) Three mutated sequences of *miR-3906* binding sites at the 3′UTR of *homer-1b* mRNA were shown at positions M1, M2 and M3. **Figure S4.** The expression patterns of *homer-1b* mRNA at various developmental stages. (**A**) Using reverse transcription-PCR to detect *homer-1b* transcripts in embryos at various stages as indicated. The *homer1b* cDNA was detected from 20 hpf until at least 72 hpf. No *homer-1b* primers were added to serve as negative control. Detection of *β-actin* cDNA served a positive control. Using whole mount *in situ* hybridization to detect the expression patterns of *homer-1b* mRNA in the muscle region of zebrafish embryos at indicated stages. (**B**–**F**) were lateral view and (**B’**, **G**) were dorsal view. The *homer-1b* was starting to express at the dorsal somite (ds) in the front of mature somites at 20 hpf (**B**, **B'**, arrow). At 22 hpf, *homer-1b* was detected at dorsal region (dorsal somite, ds) and ventral region (ventral somite, vs) in the front of mature somites (**C**, arrow). At 26 hpf, *homer-1b* expression was increased in the newly formed somites in tail (**D**). At 36 hpf, *homer-1b* transcripts were detected in trunk muscles (**E**, **F**). (**G**) At 60 hpf, *homer-1b* was also expressed in the trunk migratory muscles of fin bud (fb), posterior hypoaxial muscle (phm) and sternohyoideus (sh), as well as craniofacial muscles of adductor hyoideus (ah), adductor operculi (ao), dilator operculi (do) and levator arcus palatini (lap). **Figure S5.** Specific inhibition of *homer-1b-*morpholino (MO). (**A**) Diagrams depict the target sequences of *homer-1b*-MO against *homer-1b* mRNA fused with *egfp* mRNA (*homer-1b-*MO-*target-egfp* mRNA) and the mutated *homer-1b* mRNA at wobble sequences fused with *egfp* mRNA (*wobble homer-1b-egfp* mRNA). (**B**) The GFP signal was observed in the embryos at 32 hpf when injected with *egfp* mRNA only (100 pg), (**C**) *homer-1b*-MO (2 ng) injected with *egfp* mRNA (100 pg), (**D**) *homer-1b*-MO injected with *homer-1b*-MO-*target-egfp* mRNA, (**E**) *homer-1b*-MO injected with wobble *homer-1b-egfp* mRNA, **(F)** Photo-*homer-1b*-MO injected with *homer-1b*-MO-*target-egfp* mRNA without UV treatment and **(G)** Photo-*homer-1b*-MO injected with *homer-1b*-MO-*target-egfp* mRNA under UV exposure. GFP was not observed in embryos injected with *homer-1b*-MO and *homer-1b*-MO-*target-egfp* mRNA (**D**) and Photo-*homer-1b*-MO injected with *homer-1b*-MO-*target-egfp* mRNA under UV exposure **(E)**. **Figure S6.** Induction to produce excessive *homer-1b* at late stage caused the disarrangement of actin filaments. We microinjected plasmid pHsp-*wob-homer-1b* into one-cell wild-type embryos and heat-shocked at 20 hpf for 30 min to overexpress wobble *homer-1b* mRNA. After heat shock, we employed phalloidin staining for embryos at 32 hpf to examine the alignment of sarcomeric actin of fast muscle cells. Compared with WT (**A**), the morphological trait of embryos injected pHsp-*wob-homer-1b* (**B**) was similar to that of WT. In WT embryo **(C, C’),** the actin filaments were bent and disordered, and the boundaries of sarcomeres were vague in pHsp-*wob-homer-1b*-injected embryos **(D, D’)**. This evidence demonstrated that overexpression of *homer-1b* both at the one-celled stage and at 20 hpf caused the same effects on the arrangement of actin filaments in embryos.
(DOC)

File S2 **File containing Movie S1.** Movie S1. Swimming defect of *miR-3906*-MO and *homer-1b* mRNA injected embryos.
(AVI)
