# Supplementary material for: MicroRNA-3906 Regulates Fast Muscle Differentiation through Modulating the Target Gene homer-1b in Zebrafish Embryos
Source: PLoS One. 2013 Jul 31;8(7):e70187. doi: 10.1371/journal.pone.0070187 (PMC3729524; doi:10.1371/journal.pone.0070187)
Supplement: File S1 — File containing Figures S1–S6. Figure S1. miR-3906 persisted in the trunk muscle of zebrafish embryos at later developmental stages. (A, D) Using WISH to detect the expression of miR-3906, (B, E) miR-206 and (C, F) myf5 intron1 control-22nt of zebrafish embryos at 48 hpf and 8 dpf. (A) miR-3906 was detected both at 48 hpf and (D) 8 dpf in the trunk muscles of zebrafish embryos. (B, E) Detecting miR-206 expression served as a positive control, (C, F) while detecting the expression of myf5 intron1 control-22nt served as negative control. Figure S2. The putative target genes of miR-3906 using LAMP assay and microarray analysis. LAMP assay was performed for the cell extracts from zebrafish embryos at 32 hpf, and the possible target-RNAs of miR-3906 were analyzed by Zebrafish Whole Genome Microarray (Aligent). After standardization, 632 possible target genes of miR-3906 were predicted. Among them, 150 genes with higher binding capacity were selected and the NCBI’s ZFIN database used to define these plausible genes. Only 50 out of 150 genes had a complete gene sequence and were defined, including, for example, 4 genes of brain, 3 of the central nervous system (CNS), 1 of lens, 12 of muscles and 30 others from nonspecific regions. NA: not available. Figure S3. The mutated sequences of miR-3906 binding sites at the 3′UTR of homer-1b mRNA. (A) Three miR-3906 binding sequences at the 3′UTR of homer-1b mRNA were predicted at positions 1, 2 and 3. (B) Three mutated sequences of miR-3906 binding sites at the 3′UTR of homer-1b mRNA were shown at positions M1, M2 and M3. Figure S4. The expression patterns of homer-1b mRNA at various developmental stages. (A) Using reverse transcription-PCR to detect homer-1b transcripts in embryos at various stages as indicated. The homer1b cDNA was detected from 20 hpf until at least 72 hpf. No homer-1b primers were added to serve as negative control. Detection of β-actin cDNA served a positive control. Using whole mount in situ hybridization to [file pone.0070187.s001.doc]

**Supporting Information**


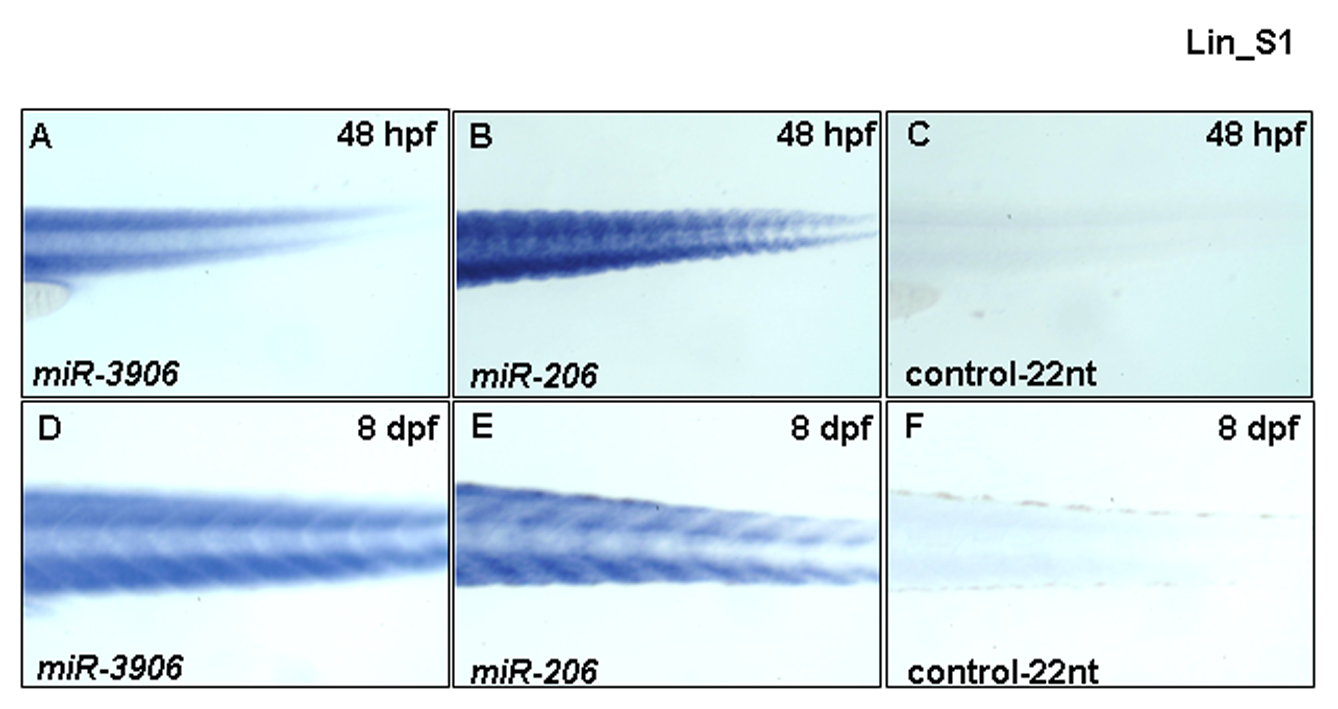
**Figure S1. *miR-3906* persisted in the trunk muscle of zebrafish embryos at later developmental stages.** (**A**, **D**) Using WISH to detect the expression of *miR-3906*, (**B**, **E**) *miR-206* and (**C**, **F**) *myf5* intron1 control-22nt of zebrafish embryos at 48 hpf and 8 dpf. (**A**) *miR-3906* was detected both at 48 hpf and (**D**) 8 dpf in the trunk muscles of zebrafish embryos. (**B**, **E**) Detecting *miR-206* expression served as a positive control, (**C**, **F**) while detecting the expression of *myf5* intron1 control-22nt served as negative control.

**
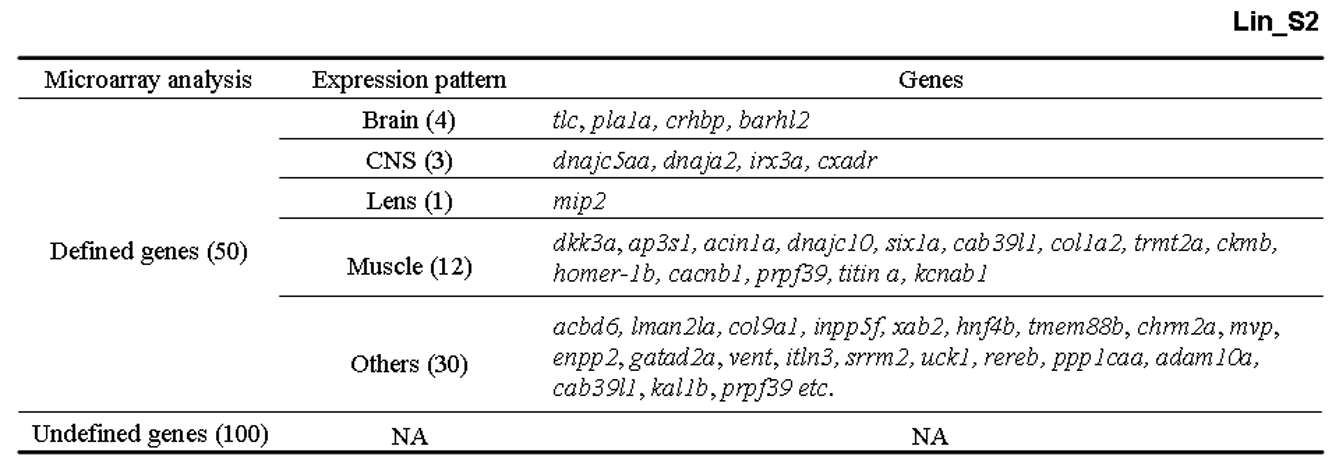
**

**Figure S2. The putative target genes of *miR-3906* using LAMP assay and microarray analysis.** LAMP assay was performed for the cell extracts from zebrafish embryos at 32 hpf, and the possible target-RNAs of *miR-3906* were analyzed by Zebrafish Whole Genome Microarray (Aligent). After standardization, 632 possible target genes of *miR-3906* were predicted. Among them, 150 genes with higher binding capacity were selected and the NCBI’s ZFIN database used to define these plausible genes. Only 50 out of 150 genes had a complete gene sequence and were defined, including, for example, 4 genes of brain, 3 of the central nervous system (CNS), 1 of lens, 12 of muscles and 30 others from nonspecific regions. NA: not available.


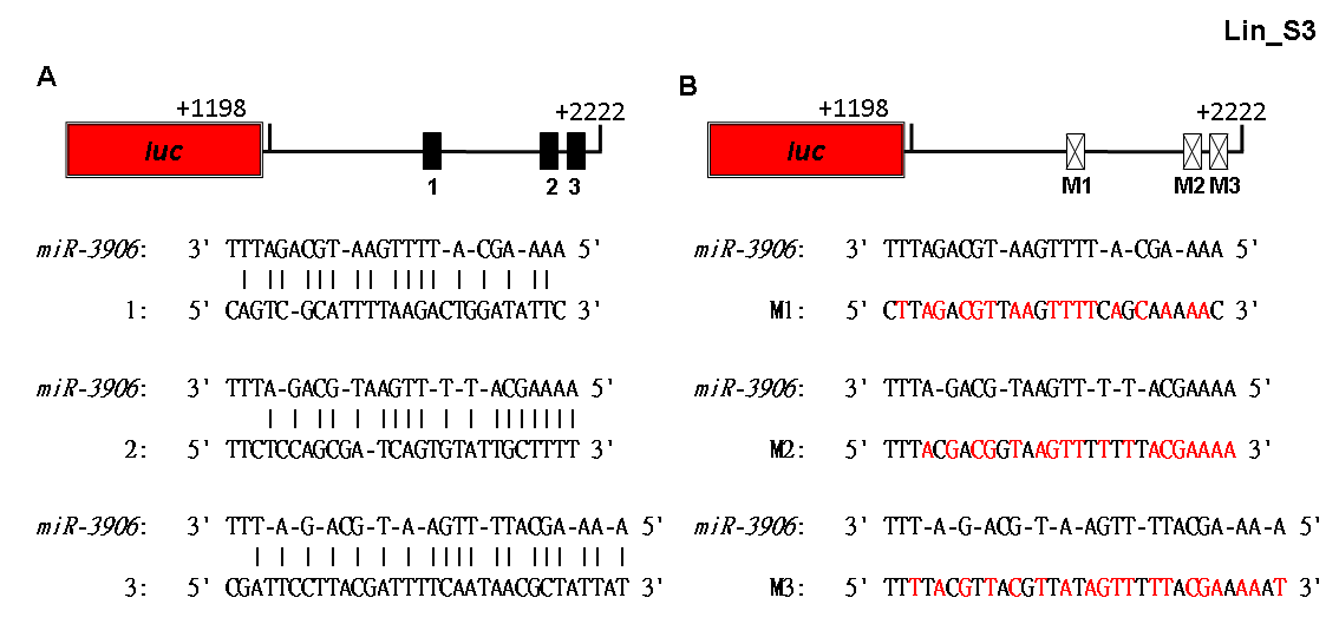


**Figure S3. The mutated sequences of *miR-3906* binding sites at the 3’UTR of *homer-1b* mRNA.** (**A**) Three *miR-3906* binding sequences at the 3’UTR of *homer-1b* mRNA were predicted at positions 1, 2 and 3. (**B**) Three mutated sequences of *miR-3906* binding sites at the 3’UTR of *homer-1b* mRNA were shown at positions M1, M2 and M3.


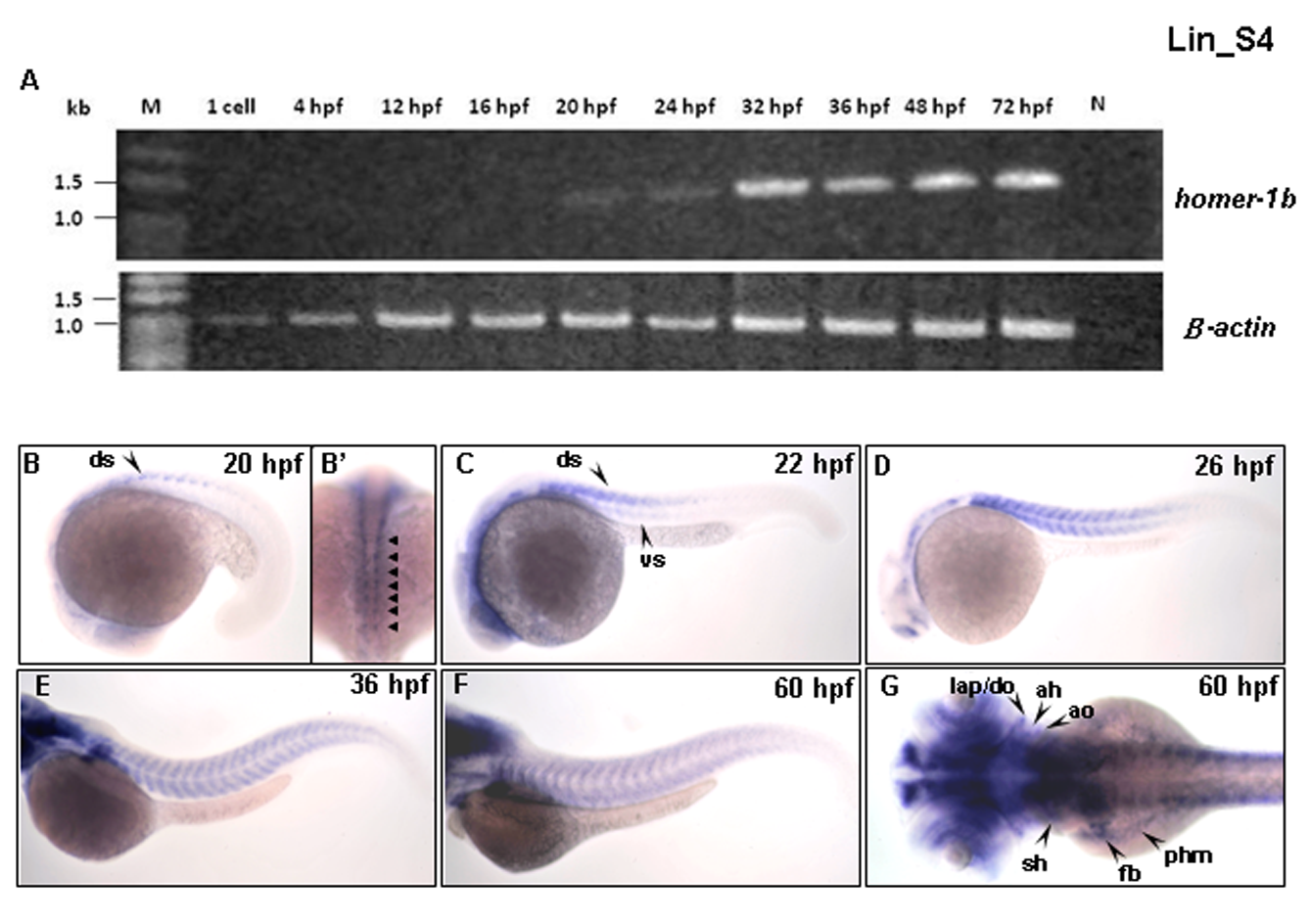


**Figure S4. The expression patterns of *homer-1b* mRNA at various developmental stages.** (**A**) Using reverse transcription-PCR to detect *homer-1b* transcripts in embryos at various stages as indicated. The *homer1b* cDNA was detected from 20 hpf until at least 72 hpf. No *homer-1b* primers were added to serve as negative control. Detection of *β-actin* cDNA served a positive control. Using whole mount *in situ* hybridization to detect the expression patterns of *homer-1b* mRNA in the muscle region of zebrafish embryos at indicated stages. (**B**-**F**) were lateral view and (**B’**, **G**) were dorsal view. The *homer-1b* was starting to express at the dorsal somite (ds) in the front of mature somites at 20 hpf (**B**, **B'**, arrow). At 22 hpf, *homer-1b* was detected at dorsal region (dorsal somite, ds) and ventral region (ventral somite, vs) in the front of mature somites (**C**, arrow). At 26 hpf, *homer-1b* expression was increased in the newly formed somites in tail (**D**). At 36 hpf, *homer-1b* transcripts were detected in trunk muscles (**E**, **F**). (**G**) At 60 hpf, *homer-1b* was also expressed in the trunk migratory muscles of fin bud (fb), posterior hypoaxial muscle (phm) and sternohyoideus (sh), as well as craniofacial muscles of adductor hyoideus (ah), adductor operculi (ao), dilator operculi (do) and levator arcus palatini (lap).

**
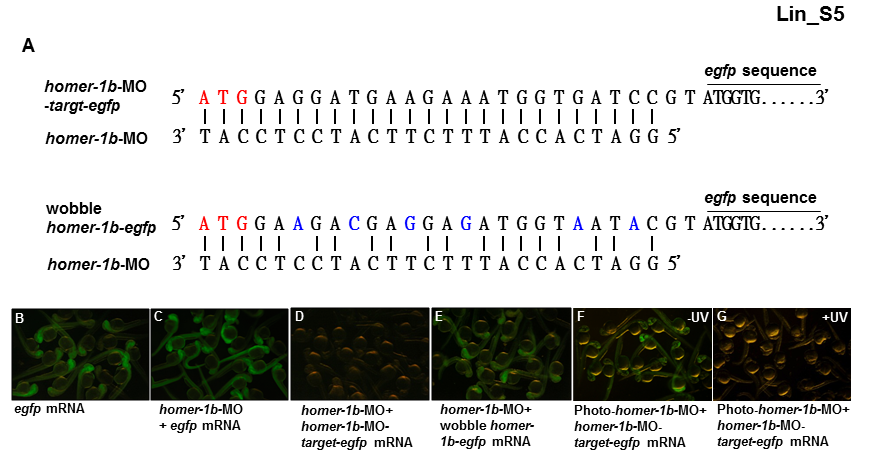
**

**Figure S5. Specific inhibition of *homer-1b-*morpholino (MO).** (**A**) Diagrams depict the target sequences of *homer-1b*-MO against *homer-1b* mRNA fused with *egfp* mRNA (*homer-1b-*MO-*target-egfp* mRNA) and the mutated *homer-1b* mRNA at wobble sequences fused with *egfp* mRNA (*wobble homer-1b-egfp* mRNA). (**B**) The GFP signal was observed in the embryos at 32 hpf when injected with *egfp* mRNA only (100 pg), (**C**) *homer-1b*-MO (2 ng) injected with *egfp* mRNA (100 pg), (**D**) *homer-1b*-MO injected with *homer-1b*-MO-*target-egfp* mRNA, (**E**) *homer-1b*-MO injected with wobble *homer-1b-egfp* mRNA, **(F)** Photo-*homer-1b*-MO injected with *homer-1b*-MO-*target-egfp* mRNA without UV treatment and **(G)** Photo-*homer-1b*-MO injected with *homer-1b*-MO-*target-egfp* mRNA under UV exposure. GFP was not observed in embryos injected with *homer-1b*-MO and *homer-1b*-MO-*target-egfp* mRNA (**D**) and Photo-*homer-1b*-MO injected with *homer-1b*-MO-*target-egfp* mRNA under UV exposure **(E)**.

**
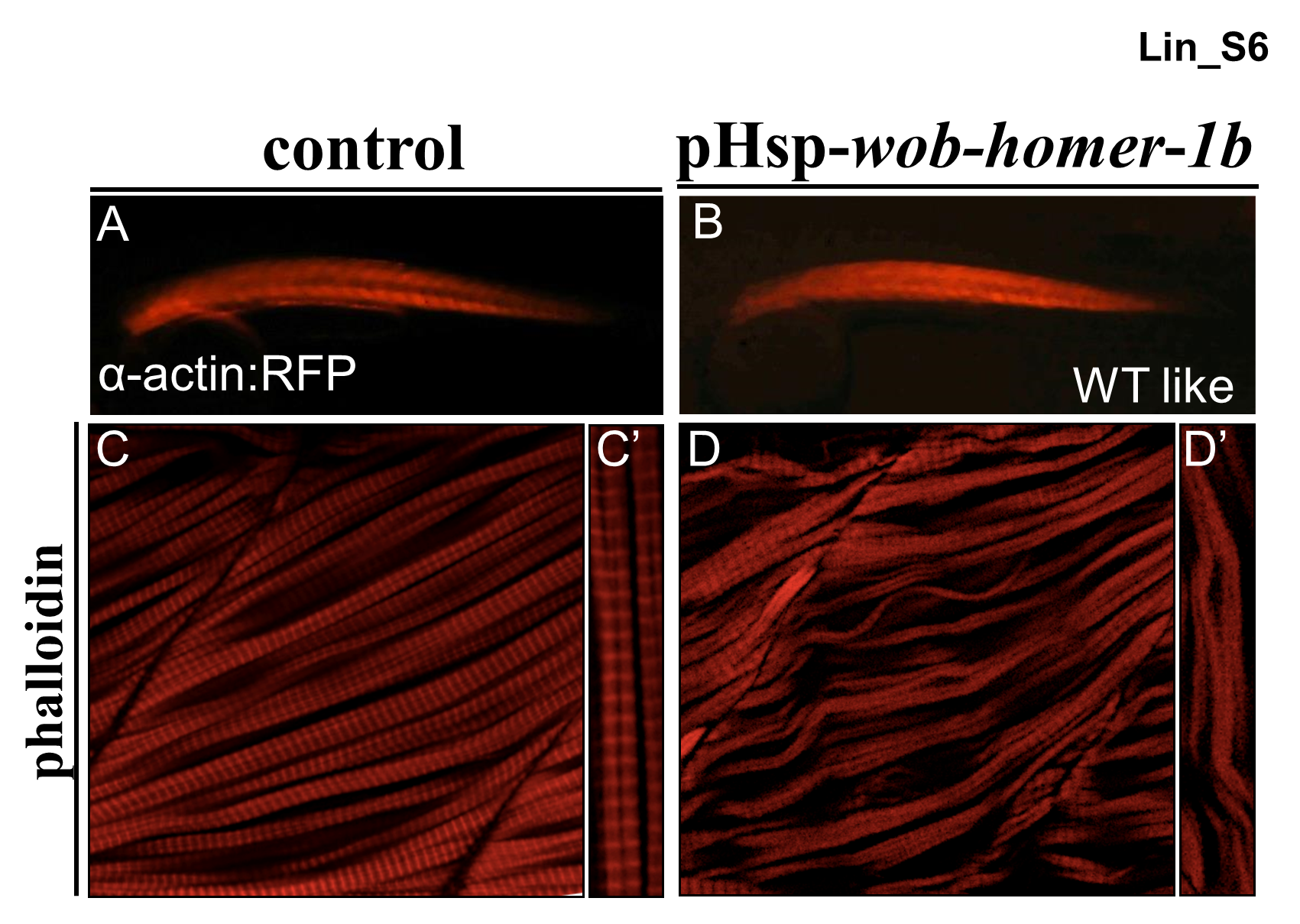
**

**Figure S6. Induction to produce excessive *homer-1b* at late stage caused the disarrangement of actin filaments.** We microinjected plasmid pHsp-*wob-homer-1b* into one-cell wild-type embryos and heat-shocked at 20 hpf for 30 min to overexpress wobble *homer-1b* mRNA. After heat shock, we employed phalloidin staining for embryos at 32 hpf to examine the alignment of sarcomeric actin of fast muscle cells. Compared with WT (**A**), the morphological trait of embryos injected pHsp-*wob-homer-1b* (**B**) was similar to that of WT. In WT embryo **(C, C’),** the actin filaments were bent and disordered, and the boundaries of sarcomeres were vague in pHsp-*wob-homer-1b*-injected embryos **(D, D’)**. This evidence demonstrated that overexpression of *homer-1b* both at the one-celled stage and at 20 hpf caused the same effects on the arrangement of actin filaments in embryos.
